# Genotyping by sequencing reveals the interspecific *C*. *maxima / C*. *reticulata* admixture along the genomes of modern citrus varieties of mandarins, tangors, tangelos, orangelos and grapefruits

**DOI:** 10.1371/journal.pone.0185618

**Published:** 2017-10-05

**Authors:** Amel Oueslati, Amel Salhi-Hannachi, François Luro, Hélène Vignes, Pierre Mournet, Patrick Ollitrault

**Affiliations:** 1 Laboratoire de Génétique Moléculaire, Immunologie et Biotechnologie, Faculté des Sciences de Tunis (FST), Université de Tunis El Manar, Tunis, Tunisia; 2 AGAP Research Unit, Centre de coopération Internationale en Recherche Agronomique pour le Développement Petit-Bourg, Guadeloupe, France; 3 AGAPResearch Unit, Institut National de la Recherche Agronomique, San Giuliano, France; 4 AGAP Research Unit, Centre de coopération Internationale en Recherche Agronomique pour le Développement, Montpellier, France; United States Department of Agriculture, UNITED STATES

## Abstract

The mandarin horticultural group is an important component of world citrus production for the fresh fruit market. This group formerly classified as *C*. *reticulata* is highly polymorphic and recent molecular studies have suggested that numerous cultivated mandarins were introgressed by *C*. *maxima* (the pummelos). *C*. *maxima* and *C*. *reticulata* are also the ancestors of sweet and sour oranges, grapefruit, and therefore of all the “small citrus” modern varieties (mandarins, tangors, tangelos) derived from sexual hybridization between these horticultural groups. Recently, NGS technologies have greatly modified how plant evolution and genomic structure are analyzed, moving from phylogenetics to phylogenomics. The objective of this work was to develop a workflow for phylogenomic inference from Genotyping By Sequencing (GBS) data and to analyze the interspecific admixture along the nine citrus chromosomes for horticultural groups and recent varieties resulting from the combination of the *C*. *reticulata* and *C*. *maxima* gene pools. A GBS library was established from 55 citrus varieties, using the ApekI restriction enzyme and selective PCR to improve the read depth. Diagnostic polymorphisms (DPs) of *C*. *reticulata/C*. *maxima* differentiation were identified and used to decipher the phylogenomic structure of the 55 varieties. The GBS approach was powerful and revealed 30,289 SNPs and 8,794 Indels with 12.6% of missing data. 11,133 DPs were selected covering the nine chromosomes with a higher density in genic regions. GBS combined with the detection of DPs was powerful for deciphering the “phylogenomic karyotypes” of cultivars derived from admixture of the two ancestral species after a limited number of interspecific recombinations. All the mandarins, mandarin hybrids, tangelos and tangors analyzed displayed introgression of *C*. *maxima* in different parts of the genome. *C*. *reticulata/C*. *maxima* admixture should be a major component of the high phenotypic variability of this germplasm opening up the way for association studies based on phylogenomics.

## Introduction

Citrus is the most important fruit crop in the world, with a production of over 156 million tons and a cultivated area of 9.8 million hectares (http://www.fao.org/faostat/en/#data, 2014). Among the commercial citrus fruits, mandarins are the second most important citrus horticultural group worldwide (46 million tons), after sweet oranges (79 million tons). ‘Mandarin’ is a common name given to most small, easy-peeling citrus fruits. Mandarin germplasm was classified as *C*. *reticulata* Blanco by Swingle and Reece [1] and Mabberley [2]. Webber [3] classed mandarin genotypes in four different groups: king, satsuma, mandarin, and tangerine. Tanaka [4] divided mandarins into five groups that included 36 species, based on morphological differences in the tree, leaves, flowers, and fruits. The genetic and cytogenetic diversity of this group, revealed by molecular markers [5–10] and chromosomal banding patterns [11], is large. It also displays a wide phenotypic diversity for fruit pomology, peel and leaf oils contents [12–13], and tolerance to biotic and abiotic stresses [14].This phenotypic and genetic variability reflects a long history of cultivation, in which many mutations and natural hybridizations, including introgression of *C*. *maxima* (Burm.) Merr. genome fragments [15–17], have given rise to the existing diversity. Modern breeding programs have also contributed to interspecific admixture within the horticultural mandarin-like group [18]. Mandarin x sweet orange and mandarin x grapefruit controlled hybridization gave rise to tangors and tangelos, respectively. Then, mandarin x tangor, mandarin x tangelo and tangor x tangelo hybridizations were carried out [18].

Citrus species are primarily diploids (2 n = 2 x = 18) and were domesticated in Southeast Asia several thousand years ago. Four ancestral taxa are recognized at the origin of all cultivated citrus [7, 16, 17, 19, 20, 21]: *C*. *maxima*, the pummelos, *C*. *medica* L., the citrons, *C*. *reticulata*, the mandarins, and *C*. *micrantha* Wester a wild citrus from the Papeda group. The differentiation between these sexually compatible taxa results from a foundation effect in four geographic zones and an initial allopatric evolution. Pummelos originated in the Malay Archipelago and Indonesia, *C*. *micrantha* in the Philippine, citrons evolved in northeastern India and the nearby region of Burma and China, and mandarins were diversified over a region including Vietnam, southern China, and Japan [4, 22, 23]. So called secondary species (*C*. *sinensis* (L.) Osb.–sweet oranges-; *C*. *aurantium* L.–sour oranges-; *C*. *paradise* Macf.–grapefruits-; *C*. *limon* (L.) Burm. -lemons-; *C*. *aurantifolia* (Christm.) Swing. -limes-) result from reticulation events between these four ancestral taxa followed by a few interspecific recombinations. Then, facultative apomixis (nucellar polyembryony) and horticultural vegetative propagation methods fixed these interspecific heterozygous structures. In particular, very important horticultural groups, such as sweet and sour oranges, grapefruit [19, 20, 16, 17], but also modern mandarins [15, 16, 17], and therefore tangors and tangelos result from admixtures between the *C*. *maxima* and *C*. *reticulata* gene pools.

A major part of the phenotypic diversity of edible citrus results from the initial differentiation between the basic taxa [24–27] and the interspecific mosaic structure is a key component driving the ideotype of the secondary species. Deciphering the interspecific admixture structure of citrus germplasm is therefore essential for efficient utilization of citrus biodiversity in innovative breeding schemes.

NGS technologies have greatly modified how plant evolution is analyzed, moving from phylogenetics to phylogenomics, based on analysis of whole genome variability. New insights have been provided into the domestication history of several fruit crops [28, 29] and cereals [30, 31]. The release of the first high quality citrus reference genome by the International Citrus Genome Consortium (ICGC) [15] implemented from a haploid clementine was a fundamental step in developing phylogenomics in citrus. Re-sequencing WGS data revealed the origin of sour orange, sweet orange and clementine [15, 32] and unexpected *C*. *maxima* introgressions in traditional mandarin genomes [15]. Moreover, the interspecific mosaic between *C*. *maxima* and *C*. *reticulata* of these varieties was deciphered along the whole citrus genome by Wu et al. [15]. The availability of a reference sequence open the way for large projects involving genotyping by whole genome re-sequencing (WGS) [33, 34]. However, WGS remains costly compared to methods of reduced genome representation sequencing, such as genotyping by sequencing (GBS) [35], or restriction-site associated DNA sequencing (RADseq) [36, 37, 38, 39]. These methods allow deep coverage of the regions adjacent to restriction sites and offer great potential for efficiently sampling entire genomes for phylogenetically informative variation. They also remain more adapted to the analysis of large segregating progenies and marker trait association studies based on linkage disequilibrium [40–46].

The objective of the present work was to develop an efficient GBS approach in citrus, to analyze in depth the phylogenomic structures of modern varieties of the horticultural groups derived from the *C*. *reticulata* and *C*. *maxima* gene pools. It concern sweet and sour oranges, grapefruits, orangelos and the “small citrus” group including the mandarins, the tangors, the tangelos and their hybrids. To that end, 55 citrus accessions were analyzed by GBS in a single Illumina Hiseq 2000 line using ApeKI as the restriction enzyme. A workflow for the identification of diagnostic polymorphisms (DPs; SNPs or Indels) of the differentiation between the two ancestral taxa was implemented. These DPs were used for the phylogenomic analysis of chromosome segments along the whole genome of the 55 varieties.

## Material and method

### Plant material

We adopted the Swingle and Reece [1] botanical classification for scientific names. In all, 55 citrus varieties from the collection of the “CRB Citrus” biological resource center managed by INRA and CIRAD in Corsica (France) were analyzed (S1 Table). Eleven accessions representative of the mandarin horticultural group and six representative of the pummelo horticultural group were selected to identify diagnostic markers of *C*. *maxima/C*. *reticulata* differentiation. Thirty-eight varieties assumed to derive from the admixture of these two taxa were included in the study (five mandarin hybrids, seven tangors (*C*. *reticulata x C*. *sinensis)*, twelve tangelos *(C*. *reticulata X C*. *paradisi)*, seven tangelo hybrids, two assumed orangelos (*C*. *sinensis X C*. *paradisi*), two grapefruits (*C*. *paradisi*), one sour orange (*C*. *aurantium*), one clementine (*C*. *clementina* but in fact a tangor) and one sweet orange (*C*. *sinensis*)). The last three varieties were among those described by Wu et al. [15] and were useful for validating our approach for the phylogenomic analysis.

### GBS analysis

#### Library preparation and sequencing

Genomic DNA was isolated using the Plant DNAeasy^®^ kit (Qiagen), following the manufacturer’s protocol. Genomic DNA concentration was adjusted to 20 ng/μl, and *Ape*KI GBS libraries were prepared following the protocol described by Eslhire et al. [35]. DNA of each sample (200 ng) was digested with the ApeKI enzyme (New England Biolabs, Hitchin, UK). Digestion took place at 75°C for 2 h and then 65°C for 20 min to inactivate the enzymes. The ligation reaction was completed in the same plate as the digestion, again using T4 DNA ligase enzyme (New England Biolabs, Hitchin, UK) at 22°C for 1 h and the ligase was inactivated prior to pooling the samples by holding it at 65°C for 20 min. Ligated samples were pooled and PCR-amplified in a single tube but further complexity reduction was achieved using PCR primers with one selective base (A) as per Sonah et al. [47]. Single-end sequencing was performed on a single lane of an Illumina HiSeq2000 (at the MGX platform in Montpellier, France).The Illumina Hiseq 2000 sequencing raw data are available in the NCBI SRA (Sequence Read Archive), under the study accession number: SRP109295.

#### SNPs and Indel genotype calling

The Tassel 4.0 pipeline [48] was used to call SNPs and Indels from the DNA sequence reads from the Illumina raw data (unfiltered fastq file). The Tassel 4.0 GBS pipeline identified good quality, unique, sequence reads with barcodes. These sequence tags were aligned to the *C*. *clementina* 1.0 reference genome (https://phytozome.jgi.doe.gov/pz/portal.html#!info?alias=Org_Cclementina) using Bowtie2 v2.2.6 [49]. Comparative genetic mapping [50, 51] revealed high synteny and collinearity between *C*. *maxima*, *C*. *sinensis*, *C*. *aurantium* and *C*. *clementina* (mostly *C*. *reticulata* genome). Therefore, the *C*. *clementina* reference genome can be considered as a good template for mapping sequences of citrus germplasm arising from the *C*.*reticulata/C*. *maxima* gene pools. Initial filtering was performed by removing SNP and Indel loci with more than 50% missing data, as well as those with a minor allele frequency (MAF) 0.05. A stringent filter was first used to remove and replace by missing data (N) genotypes that had been called with fewer than 5 reads/polymorphism/individual [52]. By the end we considered only the polymorphic positions with less than 30% of missing data for the 17 representatives of *C*. *reticulata* and *C*. *maxima*.

#### Identification of DPs of the *C*. *reticulata / C*. *maxima* differentiation

The search for DPs is based on the approach developed by Wu et al. [15] from WGS re-sequencing data. The goal is to identify polymorphisms differentially fixed between the two ancestral species. We used the inter-population differentiation parameter (G_ST_) defined by Nei [53; see below] as criteria for DPs selection. It is based on allelic frequencies within and between each species.

This analysis is complicated by the fact that we are not working with real ancestors but actual varieties resulting from the domestication process and recent studies have revealed interspecific introgressions in varieties previously considered as pure *C*. *reticulata* or pure *C*. *maxima* [15, 17]. Therefore, the selection of DPs requires identifying and removing such introgressed areas for the varieties used as references, in order to have a better estimation of the allelic frequencies and therefore of the differentiation parameter between the two ancestral taxa (G_STret-max_). The identification of Interspecific introgressions in the varieties representative of mandarins and pummelos was based on the analysis of the pattern of two parameters along the genome: the heterozygosity (Ho) and the similarity (Si-j; see below) of the considered variety with the centroid of mandarins and pummelo representative sets. The patterns of heterozygosity along the nine chromosomes were established from average values in successive windows of 120 polymorphisms each 40 polymorphisms (moving average).The estimation of similarity with mandarin and pummelo centroids was based on polymorphisms informative for the pummelo/mandarin differentiation (G_ST_>0.5). The patterns along the nine chromosomes were established from averages values in successive windows of 60 polymorphisms each 20 polymorphisms (moving average). It was expected that introgressed areas displayed significant discontinuity of these patterns according to the level of differentiation between the two taxa.

Allelic frequency in *C*. *reticulata* and *C*. *maxima* ancestral taxa and the differentiation between the two taxa (G_STret-max_) at each polymorphic position were then estimated considering missing data in the introgressed areas of the considered representative accessions of *C*. *reticulata* and *C*. *maxima*. The polymorphisms with G_STret-max_> = 0.9 were considered as diagnostic polymorphisms of *C*. *reticulata/C*. *maxima* differentiation.

To decipher the interspecific mosaic structure of each chromosome, the proportion of diagnostic markers that were homozygous for *C*. *reticulata*, *C*. *maxima* and heterozygous was analyzed in successive windows of 20 diagnostic markers. Genomic areas (windows of 20 DPs) with a best configuration frequency (*C*. *maxima*/C. maxima, *C*. *maxima/C*. *reticulata or C*. *reticulata/C*. *reticulata*) to second frequency ratio lower than 2, were considered as undetermined.

### Genetic parameters

The search for diagnostic SNPs and Indels of *C*. *maxima* and *C*. *reticulata* differentiation was based on the estimation of the inter-population differentiation parameter (G_ST_) defined by Nei [53]. It was performed from the estimated allele frequency of each taxon considering the same population size for each taxon to estimate the frequency of the whole population (Tot).

$$G_{ST}=\left(He_{Tot}-\left(He_{Reticulata}+He_{Maxima}\right)/2\right)/He_{Tot}$$

Where He is the expected proportion of heterozygous loci per individual (He = 1 − Σ pi^2^, pi is the frequency of a given allele in the considered population or subpopulation).

G_ST_ values ranged from zero to one. G_ST_ = 1 means that the two taxa were totally differentiated.

Observed heterozygosity (Ho), expected heterozygosity (He) and G_ST_ estimations were computed with Excel.

A neighbor-joining analysis [54] was computed using DARwin software version 5.0 [55]. Genetic dissimilarities were calculated using the usual Euclidean index:

$$d_{i-j}=\sqrt{{\sum_{1}^{K}\left(x_{ik}-x_{jk}\right)}^{2}}$$

Weighted neighbor-joining tree was computed from the dissimilarity matrix. The usual Euclidean index was also used for a Factorial Analysis using DARwin software.

Simple matching dissimilarity index (di-j) between pairs of accessions was used to estimate the average dissimilarity within and between mandarin and pummelo representative sets. It was computed using DARwin software:

$$d_{i-j}=1-1/L\sum_{l=1}^{L}m_{l}/2$$

Where di-j is the dissimilarity between units i and j, L is the number of loci, ml is the number of matching alleles for locus l.

The similarity index used to identify introgression in accessions representative of basic taxa is calculated as:

$$S_{i-j}=1-d_{i-j}$$

## Results

### Genotype calling and varietal sample diversity

**Fig 1:**
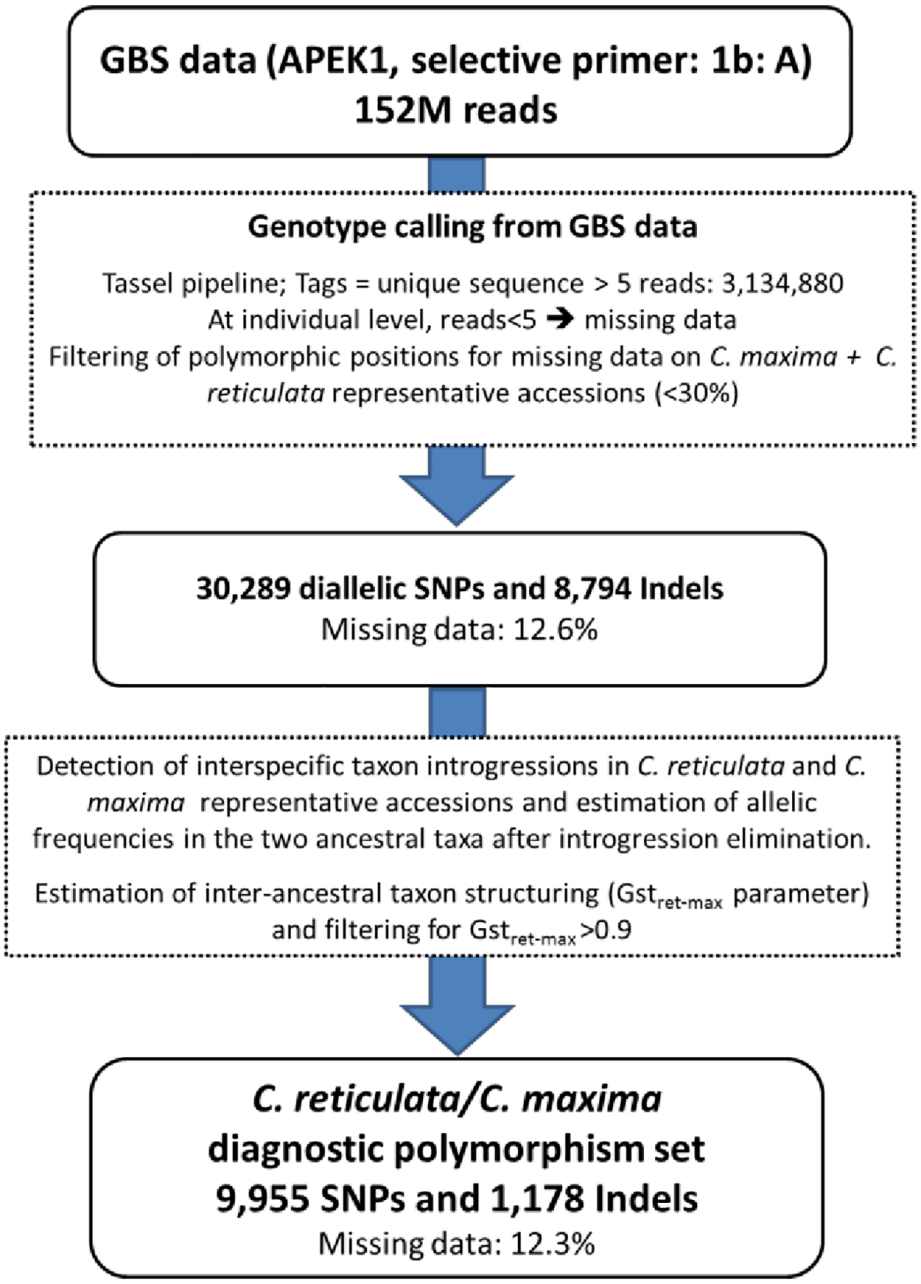
Workflow to identify diagnostic markers of *C*. *reticulata/C*. *maxima* differentiation from GBS reads.

Fifty-five varieties were sequenced in a single lane of a Hiseq 2000 (55plex) according to the Cornell GBS methodology [35] using ApeKI as the restriction enzyme and selective primer. A little more than 150 M reads were obtained. The Tassel pipeline was applied for genotype calling. Ninety-one percent of these reads were validated (bare code, restriction site plus insert) and 84.3% were mapped on the clementine reference genome [15]. Filtering with a unique sequence with at least 5 reads, 3,134,880 Tags were identified and around half of them had only one hit map on the clementine reference genome. Genotype calling from these Tags with a unique hit map was undertaken considering a position with less than five reads as missing data. Lastly, we selected only those polymorphic positions where global missing data for the representative accessions of *C*. *maxima* and *C*. *reticulata* were under 30%. In all, 30,289 SNPs and 8,794 Indels (39,083 total markers) were selected (Fig 1). For these selected genome positions, the average number of reads per individual was 34.2. The global missing data rate for this set of polymorphisms was 12.6%. 38% and 88% of the selected markers have respectively less than 5% and 25% of missing data. At individual level 33% and 93% of the varieties displayed less than 5% and 25% of missing data, respectively (S1 Fig). Three varieties (“Jakson” orangelo 68%; “Ugli” tangelo, 44% and “Mapo” tangelo, 38%) displayed a very high rate of missing data associated with a low sequencing depth.

SNP numbers varied between 2,581 on chromosome 6 and 5,440 on chromosome 3. For Indels, the range was between 735 (chromosome 1) and 1,649 (chromosome 3). The analyzed set of variety displayed a very limited default of heterozygosity according to Hardy Weinberg equilibrium (Fw = 0.083 and 0.063 for SNPs and Indels, respectively). The expected heterozygosity (i.e. equivalent to Nei genetic diversity of the population) was very similar for SNPs (0.330) and Indels (0.335) and appeared stable over the different chromosomes. Similar behavior was found for the observed heterozygosity with averages of 0.308 and 0.323 for SNPs and Indels, respectively (Table 1).

**Table 1:** Polymorphisms mined from GBS data on 55 citrus varieties along the nine chromosomes.

	SNPs				Indels			
	N	Ho	He	Fw	N	Ho	He	Fw
**C1**	2659	0.248±0.006	0.311±0.004	0.210±0.012	735	0.262±0.013	0.314±0.009	0.183±0.025
**C2**	3570	0.32±0.004	0.352±0.003	0.097±0.007	947	0.347±0.011	0.365±0.008	0.055±0.018
**C3**	5440	0.316±0.003	0.330±0.003	0.065±0.005	1649	0.33±0.009	0.344±0.007	0.070±0.015
**C4**	3034	0.297±0.002	0.333±0.002	0.102±0.004	880	0.305±0.012	0.327±0.009	0.097±0.020
**C5**	4094	0.306±0.003	0.324±0.002	0.079±0.005	1218	0.300±0.011	0.306±0.009	0.044±0.016
**C6**	2581	0.315±0.002	0.336±0.002	0.07±0.004	815	0.343±0.013	0.352±0.010	0.037±0.019
**C7**	2740	0.304±0.002	0.318±0.002	0.055±0.003	800	0.33±0.014	0.334±0.010	0.038±0.022
**C8**	2718	0.341±0.002	0.341±0.002	0.012±0.003	900	0.361±0.014	0.345±0.010	-0.017±0.019
**C9**	3453	0.313±0.002	0.321±0.002	0.073±0.004	850	0.32±0.015	0.329±0.009	0.081±0.026
**Total**	30289	0.308±0.002	0.330±0.002	0.083±0.003	8794	0.323±0.004	0.335±0.003	0.063±0.006

N: number of polymorphisms; Ho: observed heterozygosity; He: expected heterozygosity; Fw Wright fixation index; C1 to C9: the nine chromosomes

At individual level (S1 Appendix), heterozygosity varied between 0.135 (5,110 heterozygous markers) for “Cleopatra” mandarin and 0.544 (20,851 heterozygous markers) for sour orange with a total average of 0.316. The lower heterozygosity values were observed within the representatives of pummelos (average 0.150) and mandarins (average 0.217) while the secondary species *C*. *aurantium* (0.544), *C*. *sinensis* (0.490) and *C*. *paradisi* (0.476) displayed higher values. The heterozygosity of modern mandarin hybrids, tangors, tangelos, and orangelos varied between 0.235 (“Sunrise” tangelo) and 0.481 (“Pearl” tangelo) with an average of 0.359. The distribution of heterozygous loci over the nine chromosomes depended on the varieties (S1 Appendix). The smallest number of heterozygous markers (162) was observed for the “Jakson” orangelo on chromosome 6. This low value was mainly due to the high rate of missing data for this variety. Globally, for all the analyzed varieties, our GBS approach provided good coverage of the nine chromosomes with heterozygous markers.

### Diversity between mandarins and pummelos and search for DPsof *C*. *reticulata/C*. *maxima* differentiation

#### Genetic parameters

**Fig 2:**
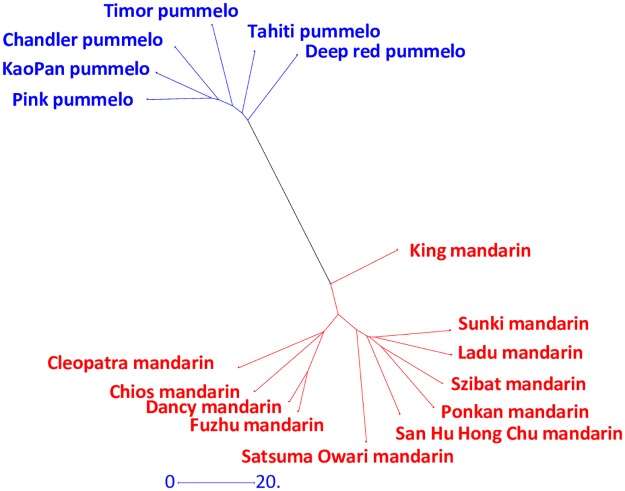
NJ tree analysis of the representatives of pummelos and mandarins.

All the analyses were based on polymorphic positions with less than 30% of missing data in the 17 *C*. *reticulata/C maxima* representative accessions. The number of polymorphic positions was 26,076, 12,682 and 37,430 in mandarins, pummelos (Table 2) and mandarins plus pummelos (Table 3), respectively. Interestingly 95.76% of the polymorphisms identified in the whole sample of 55 varieties were found in the set of 17 mandarin and pummelo representative accessions. With twice the number of polymorphic sites and higher expected heterozygosity (He = 0.184 and 0.119 for the mandarin and pummelo samples, respectively), the mandarin set appeared more polymorphic than the pummel set. Limited excess of observed heterozygosity was observed in both sets (Fw = -0.091 and -0.199 in mandarins and pummelos, respectively). The differentiation between the two representative sets was high, as revealed by the Fw and G_ST_ values (average 0.349 and 0.471 respectively), the NJ tree analysis (Fig 2), and the average dissimilarity within and between the mandarin and pummelo representative sets. Indeed the average differentiations between varieties within the mandarin and pummelo sets were respectively 0.173+/-0.012and 0.109+/-0.009 while the interspecific average dissimilarity was 0.527+/-0.010.

**Table 2:** Polymorphisms (SNPs and Indels) mined from GBS data on 11 mandarin and 6 pummelo varieties along the nine chromosomes; intra horticultural group diversity.

	Mandarin				Pummelos			
	N	Ho	He	Fw	N	Ho	He	Fw
**C1**	1517	0.167±0.008	0.141±0.006	-0.138±0.017	942	0.129±0.008	0.103±0.006	-0.22±0.027
**C2**	3077	0.231±0.007	0.210±0.005	-0.083±0.011	1618	0.151±0.007	0.129±0.005	-0.149±0.022
**C3**	5075	0.220±0.006	0.196±0.004	-0.078±0.010	2145	0.132±0.006	0.108±0.004	-0.191±0.018
**C4**	1947	0.16±0.007	0.145±0.006	-0.083±0.014	1049	0.111±0.007	0.098±0.005	-0.130±0.026
**C5**	3322	0.186±0.007	0.153±0.005	-0.121±0.012	1870	0.165±0.007	0.133±0.005	-0.212±0.021
**C6**	2664	0.241±0.007	0.213±0.005	-0.104±0.012	966	0.138±0.009	0.106±0.006	-0.262±0.025
**C7**	2663	0.192±0.007	0.177±0.005	-0.063±0.011	1202	0.139±0.008	0.121±0.006	-0.130±0.026
**C8**	2877	0.255±0.007	0.232±0.006	-0.074±0.012	1438	0.198±0.010	0.151±0.007	-0.263±0.024
**C9**	2934	0.213±0.007	0.190±0.005	-0.099±0.011	1452	0.164±0.009	0.127±0.006	-0.241±0.026
**Total**	26076	0.208±0.002	0.184±0.002	-0.091±0.004	12682	0.147±0.003	0.119±0.002	-0.199±0.008

**Table 3:** Polymorphisms (SNPs and Indels) mined from GBS data on 11 mandarin and 6 pummelo varieties along the nine chromosomes; global diversity and inter-horticultural group differentiation.

	Mandarins+Pummelos				
	N	Ho	He	Fw	G_ST_
**C1**	3395	0.156±0.006	0.392±0.005	0.459±0.019	0.573±0.013
**C2**	4517	0.204±0.005	0.373±0.004	0.339±0.015	0.446±0.010
**C3**	6670	0.192±0.004	0.355±0.004	0.339±0.013	0.465±0.008
**C4**	3686	0.144±0.005	0.358±0.005	0.449±0.018	0.540±0.012
**C5**	4975	0.182±0.005	0.350±0.005	0.346±0.016	0.478±0.010
**C6**	3239	0.206±0.005	0.366±0.005	0.32±0.017	0.468±0.011
**C7**	3342	0.175±0.005	0.351±0.006	0.367±0.017	0.460±0.012
**C8**	3448	0.239±0.006	0.351±0.005	0.229±0.016	0.372±0.010
**C9**	4158	0.199±0.005	0.360±0.005	0.305±0.017	0.452±0.011
**Total**	37430	0.189±0.002	0.361±0.002	0.349±0.005	0.471±0.004

N:numberofpolymorphisms,Ho:observedheterozygosity;He:expectedheterozygosity;Fw:Wrightfixationindex; C1 to C9: the nine chromosomes

#### Search for DPs of *C*. *reticulata/C*. *maxima* differentiation

To estimate the differentiation parameter between *C*. *maxima* and *C*. *reticulata* (G_STret-max_) at each polymorphic position we first searched for introgressed areas in the genomes of the representative varieties of these two taxa. It was based on the analysis of the pattern of two parameters along the genome: the heterozygosity and the similarity of the considered variety with the centroid of mandarins and pummelo representative sets.

**Fig 3:**
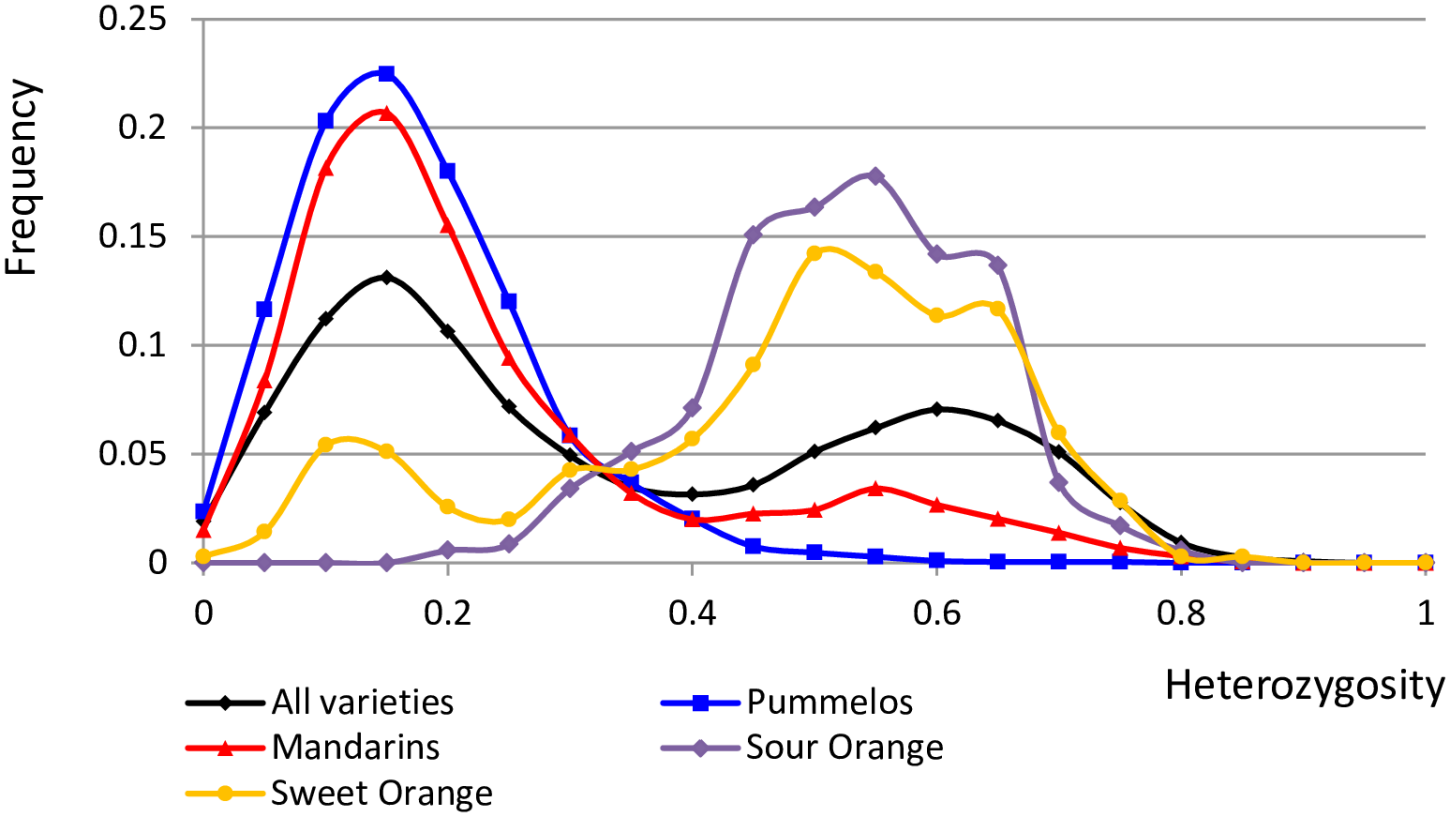
Distribution of heterozygosity computed from average values of successive windows of 100 polymorphisms along the genome. (the frequency y represent the proportion of genome fragments (windows) with Ho between x and x + 0.05).

Heterozygosity pattern discontinuity was a key for identifying introgressions in the genomes of basic taxa in the Wu et al. [15] study. These discontinuities of heterozygosity in admixed varieties were directly linked with the average differentiation between varieties at intra and inter-specific levels. They distinguished two distinct features in the nucleotide heterozygosity distribution: one averaging ~6 het sites/kb corresponding to intraspecific heterozygosity and the other ~17 het sites/kb corresponding to interspecific *C*. *reticulata/C*. *maxima* heterozygosity. For GBS data, we analyzed the heterozygosity in genomic windows covering successive set of 100 polymorphic positions along the genomes. When looking the full set of varieties, it reveals a bimodal distribution (Fig 3). The first mode and second mode correspond respectively to heterozygosity values between 0.10–015 and 0.55–0.60. Interestingly the sour orange (*C*. *aurantium*) proved to be a F1 *C*. *maxima x C*. *reticulata* interspecific hybrid [15] displays an unimodal distribution of heterozygosity with an average value of 0.55. The sweet orange displays a bimodal distribution of heterozygosity as observed by Wu et al. [15] from WGS data.

The distribution of the six pummelo representatives is mostly unimodal with an average of 0.15. However the accurate analysis of each pummelo variety along the 9 chromosomes revealed small discontinuities for heterozygosity patterns ~24.5 Mbp from the start of chromosome 2 of “Chandler” and “Kao Pan” pummelos with respective average heterozygosity values 0.511 and 0.488. The same discontinuity of heterozygosity was previously reported for Chandler from WGS data [15].

**Fig 4:**
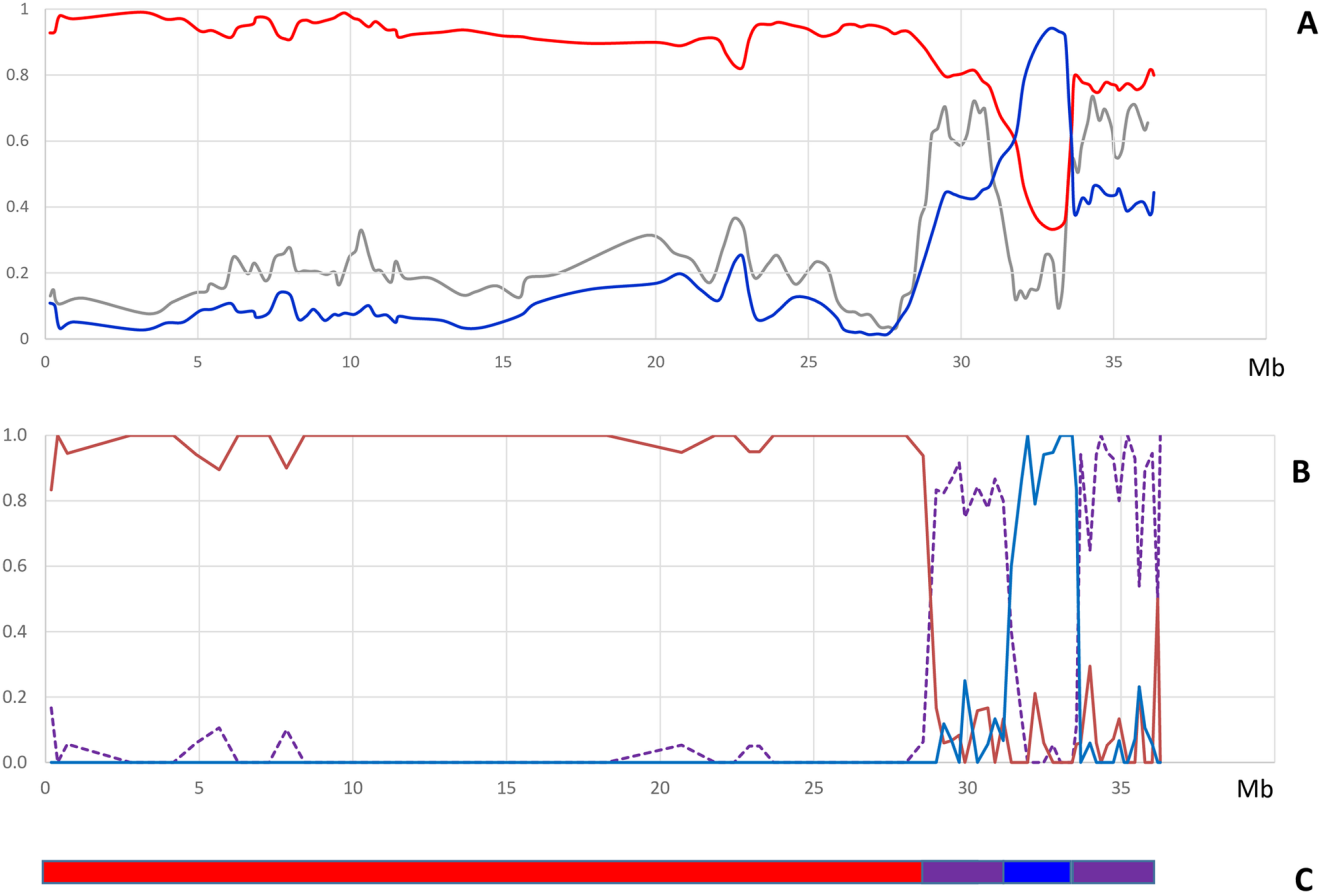
Identification of interspecific introgressions in accessions representative of ancestral taxa: Example of the “Ponkan” variety for chromosome II. A: observed heterozygosity (gray), similarity with centroids of mandarins (red) and pummelos (blue); windows of 20 markers (G_ST_1>0.5) B: proportion of *C*. *reticulata/C*. *maxima* diagnostic polymorphisms in homozygosity for *C*. *reticulata* (red), for *C*. *maxima* (blue) and interspecific heterozygosity (purple). C: deduced phylogenomic structure of Ponkan mandarin chromosome II (red: *C*. *reticulata* homozygosity; blue: *C*. *maxima* homozygosity; purple: *C*. *maxima/C*. *reticulata* heterozygosity).

The set of 11 mandarin representatives displays a major peak with a mode ~0.10–0.15 very similar to the one of the pummelo distribution. A secondary peak is observed with a mode ~0.50–0.55. Such bimodal distribution is also observed at individual level for most mandarins but also tangors, grapefruits, orangelos, tangelos and hybrids (S1 Appendix). The two modes of heterozygosity distribution fit well with the average dissimilarity values within and between the mandarin and pummelo representative sets estimated above. Moreover, these two regimes of nucleotide heterozygosity are organized along the genome as distinct blocks with clear discontinuities (Fig 4A). As previously proposed by Wu et al. [15] we considered that the regions of lower heterozygosity represent diploid segments combining two haplotypes from the same species while regions of higher heterozygosity were interpreted as hybrid segments in which the haplotype from two different species were paired. Ho values lower than 0.30 were assumed representative of intraspecific haplotype combination. We considered Ho = 0.40 as a threshold, with higher value revealing potential introgressions. These suspected introgressed areas were removed to estimate the allelic frequencies of the two ancestral taxa and the parameter (G_ST ret-max_).

**Fig 5:**
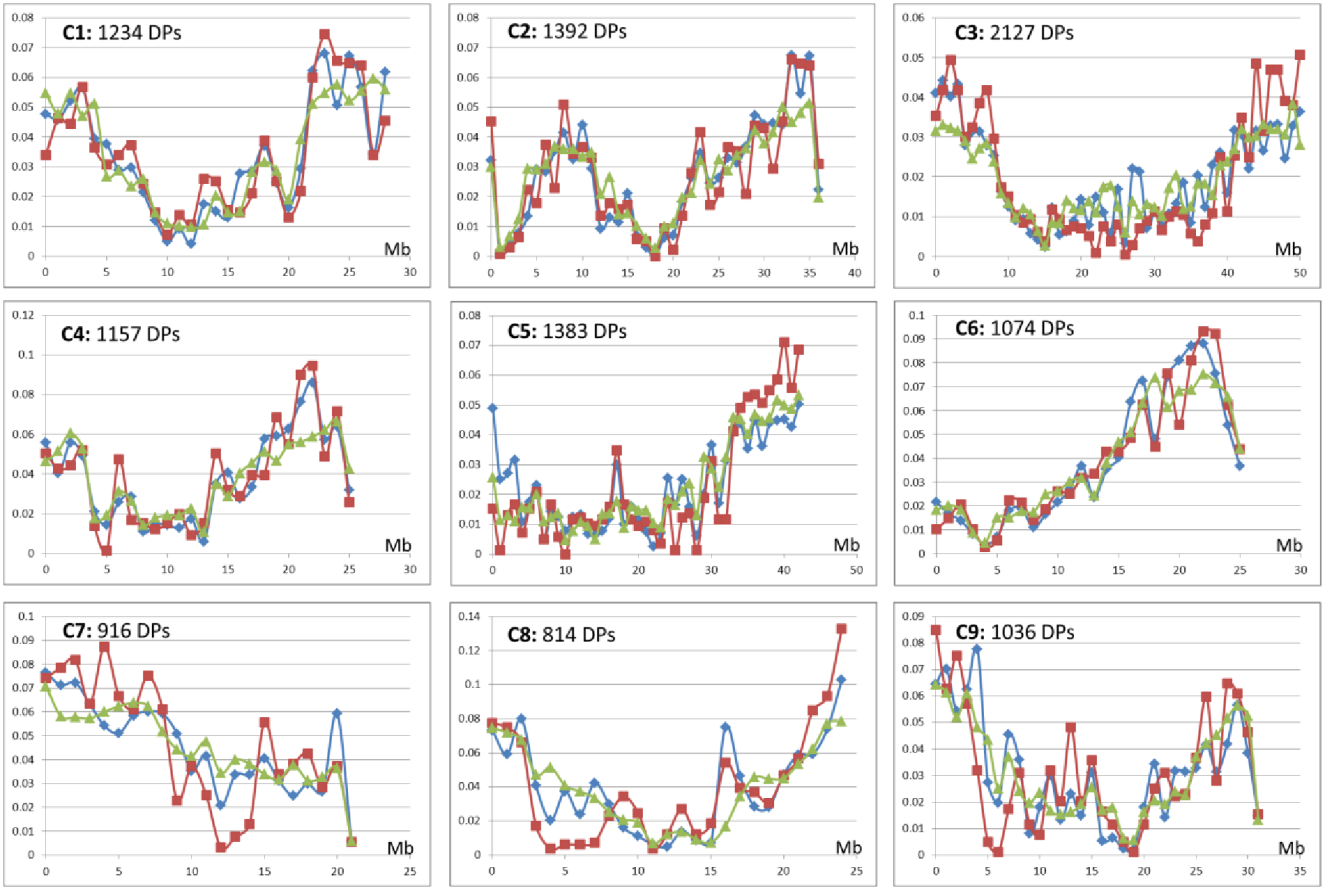
Distribution along the genome of diagnostic polymorphisms (red), whole set of polymorphisms (blue), and genes (green); relative frequency by successive windows of 1 Mb for each chromosome.

An example is given for chromosome II of the “Ponkan” mandarin (Fig 4A). *C*. *maxima* introgression in this mandarin cultivar was clearly identified at the end of the chromosome with correlated decreasing similarity with the mandarin centroid and increasing with the pummel centroid. Moreover, the Ho pattern, combined with the similarities with mandarin and pummelo centroids, suggested a succession of *C*. *reticulata/C*. *maxima* heterozygosity, *C*. *maxima* homozygosity and *C*. *reticulata/C*. *maxima* heterozygosity. All this area starting at 28,503 Mb up to the end of the chromosome was considered as potentially introgressed by *C*. *maxima*. The same analysis was carried out for the 17 accessions representative of mandarins and pummelos for the nine chromosomes. Then the allelic frequency in *C*. *reticulata* and *C*. *maxima* ancestral taxa and the differentiation between the two taxa (G_STret-max_) were estimated considering missing data in the introgressed areas. The distribution of inter-taxa G_ST_ values before and after removing introgressed areas are given in S2 Fig. After removing introgressed areas, 11,133 polymorphisms with G_ST_ over 0.9 were identified while there were only 6,867 from the initial data. These polymorphisms with G_STret-max_> 0.9 were considered as diagnostic polymorphisms of *C*. *reticulata/C*. *maxima* differentiation. To avoid redundancy of information only the first positions of Indels were considered for the following studies of the phylogenomic structure of modern varieties. Therefore, in the end, 9,955 SNP and 1,178 Indel (total 11,133) DPs were selected over the nine chromosomes (Table 4 and S2 Table) and used to infer the interspecific mosaic structures of the 55 analyzed varieties. The average missing data rate for these DPs was 12.32%. Interestingly, (Fig 5) the distribution of the 11,133 selected DPs along the genome was very similar to that of annotated genes [15] and of the whole set of mined polymorphisms (WPs).

**Table 4:** Distribution of diagnostic polymorphisms among the nine chromosomes.

	C1	C2	C3	C4	C5	C6	C7	C8	C9	Total
**TotalDPs**	1234	1392	2127	1157	1383	1074	916	814	1036	11133
**Indels**	125	141	239	111	123	132	106	98	103	1178
**SNPs**	1109	1251	1888	1046	1260	942	810	716	933	9955

DPs: diagnostic polymorphisms; C1 to C9: the nine chromosomes.

### Phylogenomic structure of modern varieties

**Fig 6:**
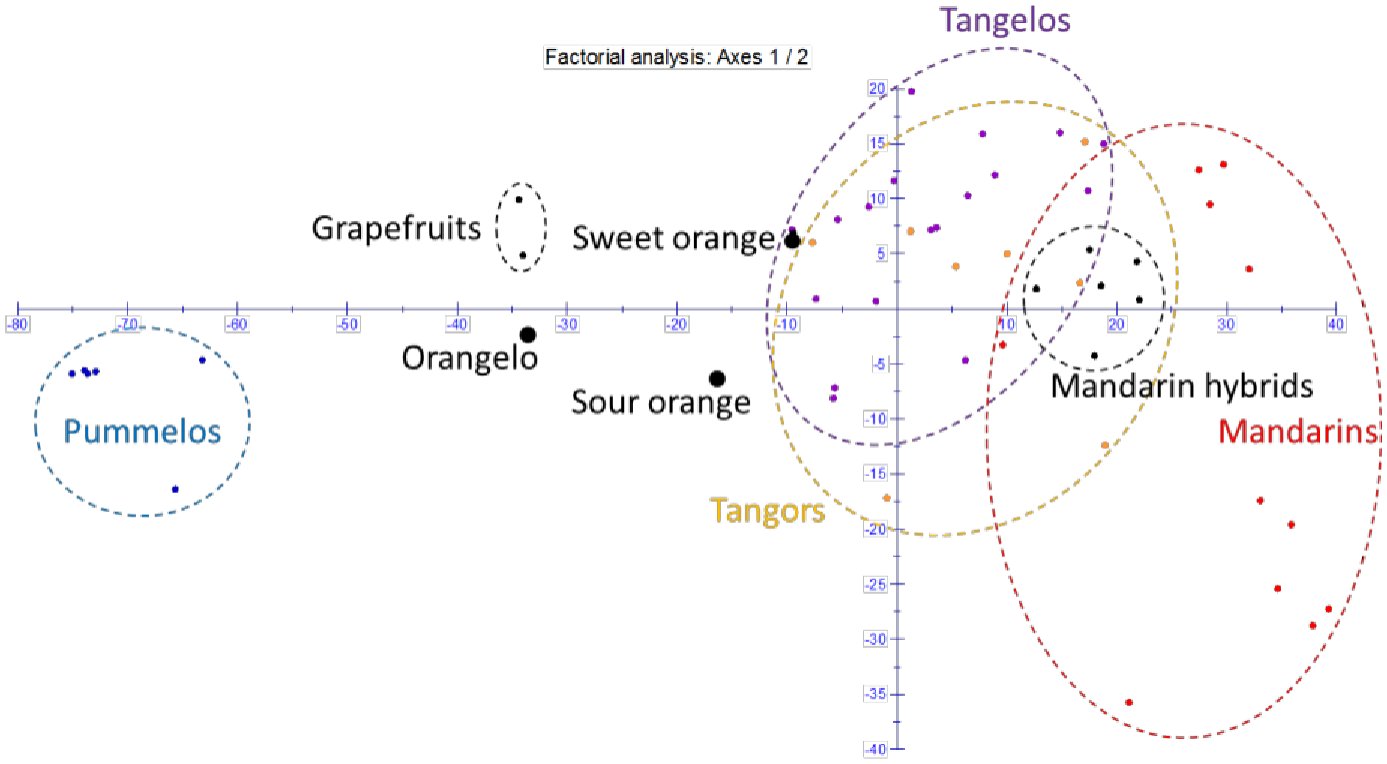
Factorial analysis from the dissimilarity table for 54 varieties, 30,943 markers.

A factorial analysis from dissimilarity table was performed to analyze the global genetic diversity organization of all the analyzed cultivars (Fig 6). The 39,083 polymorphic positions were filtered to have less than 20% of missing data over the whole sample and the “Jackson” orangelo was removed due to its very high proportion of missing data (68%). The factorial analysis was therefore carried out for 54 varieties and 30,943 markers. Pummelo/mandarin differentiation was the main structuring component defining the first axis (38.62% of total diversity). The second axis (6.47%) separated two groups of mandarin varieties according to the previous NJ analysis. The inertias of the third and fourth axes amounted to 5.03% and 3.65%, respectively. The tangor and tangelo (+tangelo hybrid) groups displayed intra-group polymorphisms and were not differentiated from each other. They still closely associated with the mandarin group but were globally slightly displaced on the pummelo side of the first axis. Sour orange and sweet orange displayed an intermediary position between the mandarin and pummelo groups, while grapefruit and orangelo (“Triumph”) were closer to the pummel cluster.

**Fig 7:**
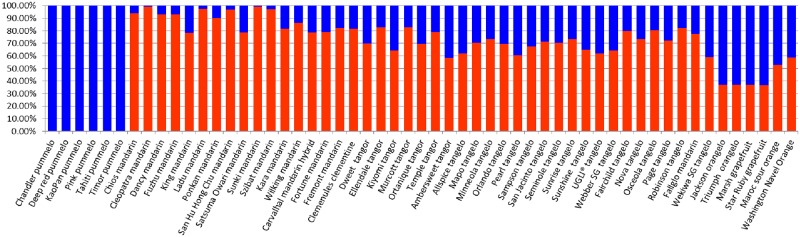
Overall proportion of *C*. *maxima* and *C*. *reticulata* admixture estimated from diagnostic polymorphisms for the 55 varieties. Red: *C*. *reticulata*; blue: *C*. *maxima*.

The proportion of *C*. *reticulata* and *C*. *maxima* admixture in the 55 varieties was estimated directly from the genotyping of the 11,133 DPs (Fig 7 and S3 Table). The six pummelos analyzed appeared to be very good representatives of *C*. *maxima* while *C*. *maxima* introgressions were found in all mandarins. However, these introgressions appeared very limited in “Cleopatra” and “Sunki” mandarins (<1%), and still low for “Ladu” and “Szibat” mandarins (<3%). Among the mandarins considered as representative of *C*. *reticulata*, the “King” mandarin and the “Satsuma” mandarins displayed the highest rates of *C*. *maxima* introgression (22% and 21%, respectively). The *C*. *maxima* proportion in tangors and tangelos (+ tangelo hybrids) ranged between 17%-“Murcott”- and 41% -“Ambersweet”- and between 19% -“Osceola”- and 41% -“Wekiwa”-, respectively). The tangor Clementine displayed 19% of *C*. *maxima* genome. The two orangelos and two grapefruits displayed a 63% *C*. *maxima* contribution. Sour orange had close to half of the two ancestors genome contribution while sweet orange displayed a 41%*C*. *maxima* contribution.

To decipher the interspecific mosaic structure of each chromosome, the proportion of diagnostic markers that were homozygous for *C*. *reticulata*, *C*. *maxima* and heterozygous was analyzed in successive windows of 20 DPs. For “Ponkan” (Fig 4B) this analysis clearly confirmed the hypothesis of successive introgression in heterozygosity and homozygosity formulated on the basis of the patterns of heterozygosity and similarities with the *C*. *reticulata* and *C*. *maxima* representative centroids (Fig 4A). The interspecific mosaic structure of “Ponkan” on C2 was inferred from this analysis (Fig 4C). This picture totally tallied with the conclusions of Wu et al. [15], established from whole genome re-sequencing data. Phylogenomic karyotypes of all the analyzed varieties were inferred in the same way.

**Fig 8:**
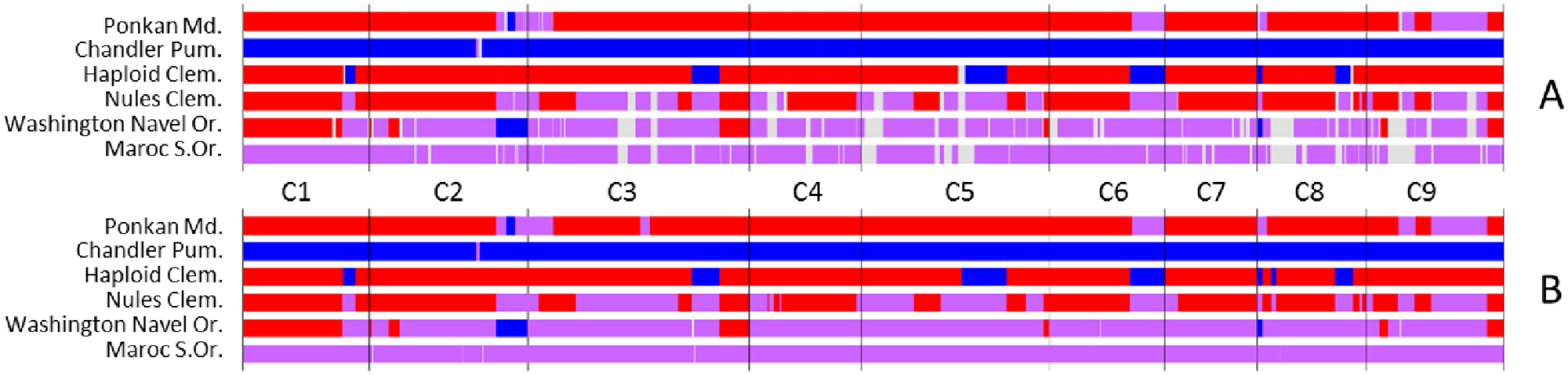
Comparison of “phylogenomic karyotypes” inferred from GBS data (A) and WGS data (B; from Wu et al. data (15)] for the “Ponkan” mandarin, the “Chandler” pummelo, the haploid clementine used to establish the citrus reference sequence, the diploid “Nules” clementine, the “Washington Navel” sweet orange and the sour orange. Red: *C*. *reticulata* homozygosity; blue: *C*. *maxima* homozygosity; purple: *C*. *reticulata/C*. *maxima* heterozygosity; grey: undetermined.

**Fig 9:**
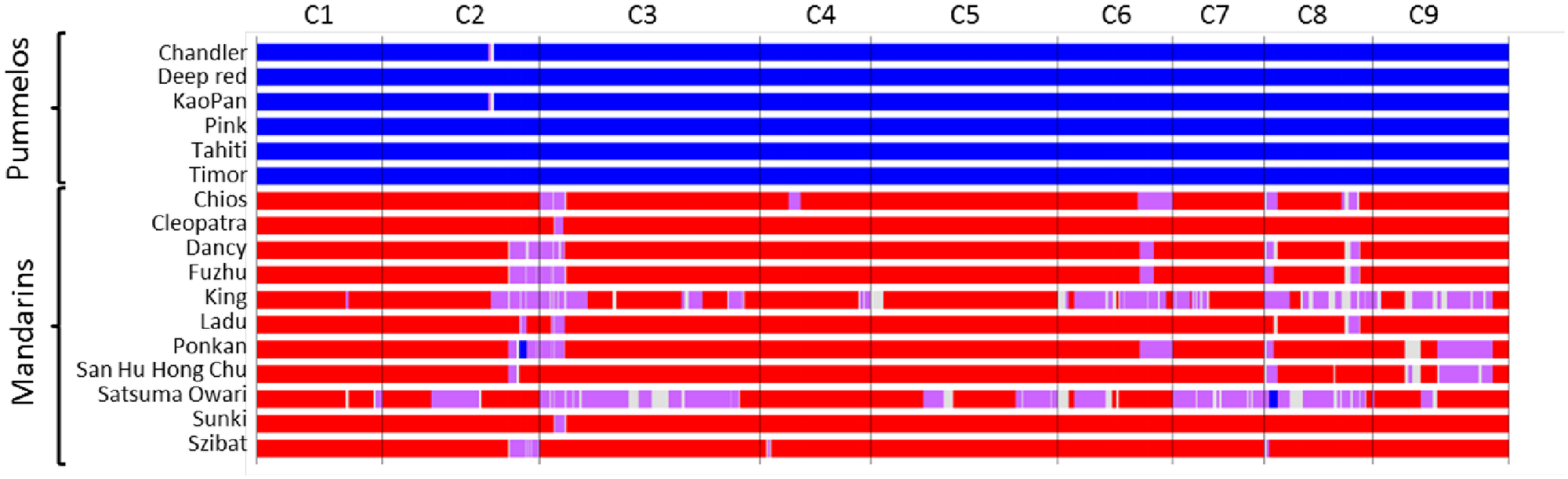
“Phylogenomic karyotypes” of the citrus accessions used as a reference for *C*. *maxima* and *C*. *reticulata* ancestral taxa. Red: *C*. *reticulata* homozygosity; blue: *C*. *maxima* homozygosity; purple: *C*. *reticulata/C*. *maxima* heterozygosity; grey: undetermined.

**Fig 10:**
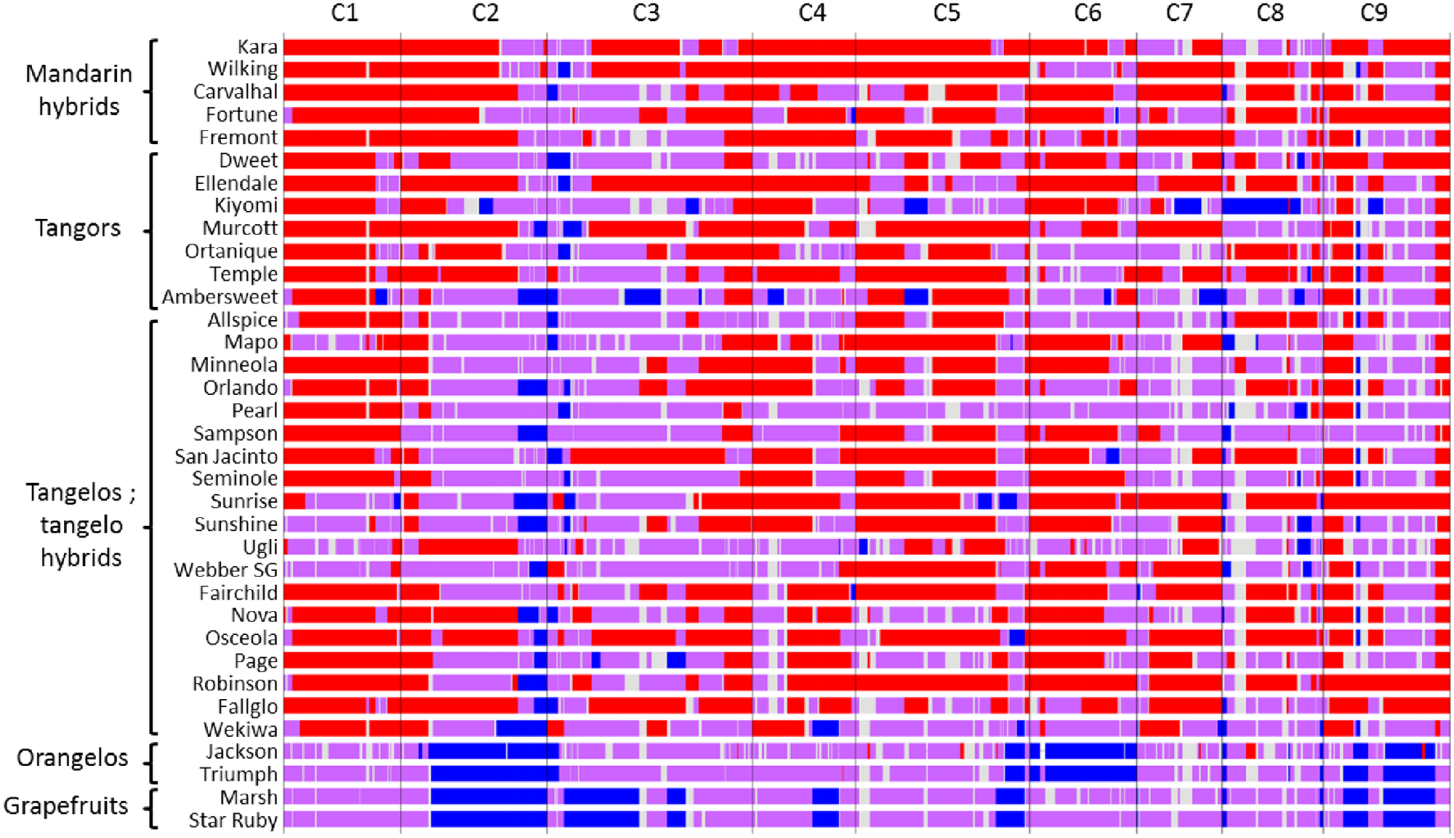
“Phylogenomic karyotypes” of 38 citrus varieties derived from the *C*.*reticulata/C*. *maxima* gene pools. Red: *C*. *reticulata* homozygosity; blue: *C*. *maxima* homozygosity; purple: *C*. *reticulata/C*. *maxima* heterozygosity; grey: undetermined.

First we compared the karyotypes inferred from GBS with that proposed by Wu et al. [15] from WGS data for the haploid clementine used to establish the citrus reference sequence, the diploid “Clemenules” clementine, the “Ponkan” mandarin, the “Chandler” pummelo, the “Washington Navel” sweet orange and the sour orange (Fig 8). For “Ponkan”, “Chandler” and the haploid clementine, no undetermined areas occurred and the karyotype totally fitted, except for two small genomic areas considered to be introgressed by *C*. *maxima* in the Wu et al. [15] study, at the beginning of C8 for the haploid clementine and in heterozygosity in the middle of C3 for “Ponkan”. In both cases, there was a very low density of DPs in these genomic areas. For the other three genotypes, common to Wu et al [15] and our study, more genome areas with undetermined origin occurred with the GBS data than WGS data. However, the conclusions totally fitted in the determinate areas. We can therefore consider that our GBS approach was validated. Among the other five representatives of *C*. *maxima*, only the “Kao Pan” pummelo displayed slight *C*. *reticulata* introgression in heterozygosity at the same location (C2) as the Chandler pummelo (Fig 9A). Therefore, modern pummelos appear to be very good representatives of *C*. *maxima*. Conversely, all the mandarins appeared introgressed by *C*. *maxima* (Fig 9B), sometimes in homozygosity as observed for “Ponkan” (C2) and “Satsuma” (C8) but mostly in heterozygosity. “Sunki” and “Cleopatra” displayed the purest *C*. *reticulata* genome with only slight *C*. *maxima* introgression on C3. “Satsuma” and “King” presented large genome areas in *C*. *maxima/C*. *reticulata* heterozygosity for eight chromosomes. Phylogenomic karyotypes were also inferred for the remaining 35 varieties (Fig 10). Phylogenomic karyotypes of grapefruits (*C*. *paradisi*) were inferred. For the identified areas, the results were identical for “Marsh and “Star Ruby” grapefruits. Their genomes appeared as mosaics of large fragments of *C*. *maxima/C*. *maxima* homozygosity and *C*. *maxima/C*. *reticulata* heterozygosity. The “Jackson” and “Triumph” orangelos displayed very similar karyotypes with patterns close to the grapefruit for chromosomes 1, 2, 5, 7, 8, and 9. Modern hybrids of mandarin, tangelos, and tangors displayed a wide range of patterns of admixture and appeared as mosaics of large fragments in *C*. *reticulata/C*. *reticulata* homozygosity, *C*. *maxima/C*. *maxima* homozygosity and *C*. *maxima/C*. *reticulata* heterozygosity. In this germplasm, *C*. *reticulata* phylogenomic homozygosity was more frequent than *C*. *maxima* homozygosity.

## Discussion

### An efficient GBS approach for citrus genotyping

With NGS development, the simultaneous discovery and genotyping of Indels and SNPs is now possible [56]. Of the different methods for reducing genome complexity to allow multiplexing of numerous samples, GBS provides the advantage of a simplified library preparation procedure from small amounts of DNA [47]. To avoid consuming too many sequence reads on highly repetitive sequences of plant genomes and provide better read mapping and genotype calling, it is important that the reduction in genome complexity be associated with a bias toward coding regions [57]. For this purpose, Elshire et al. [35] successfully used the ApeKI restriction enzyme during library preparation for barley and maize. The same favorable bias toward coding regions with ApeKI was also demonstrated in soybean [47] and *Populus* [57]. The compromise between marker density and read depth is another important issue for GBS [47, 58]. Indeed read depth average and distribution are keys for the rates of missing data and therefore the number of useful polymorphisms. Sonah et al. [47] improved the GBS approach in soybean by adding a genome complexity reduction step, using selective primers during library preparation. To obtain a good read depth and therefore an acceptable rate of missing data, we adopted the Sonah et al. [47] approach using ApeKI as the restriction enzyme and further complexity reduction using PCR primers with one selective base (A), coupled with 55plex sequencing (single end) in an Illumina HisSeq 2000. As for barley, maize [35], soybean [47] and *Populus* [57], we observed a strong link between the coding sequence distribution and the marker density along the genome. In all, 30,289 SNPs and 8,794 Indels (39,083 total markers) were selected. For these markers the average number of reads per individual was 34.2. The overall missing data rate was 12.6%. The genetic parameters evaluated were similar for SNPs and Indels and revealed greater polymorphism in mandarin representatives than pummelo accessions, as observed in previous studies with SNPs [17, 20] and Indels [59].The overall genetic organization of the whole set of varieties, provided by the factorial analysis, was globally driven by differentiation between the mandarin and pummelo representative accessions with intermediate positions of secondary species (*C*. *sinensis*, *C*. *aurantium*,*C*. *paradisi*) and all the modern varieties of mandarin hybrids, tangelos, tangors and orangelos. Such a picture is in full agreement with previous molecular studies [6, 7, 17, 19, 20] demonstrating that this germplasm resulted from admixture between the *C*. *maxima* and *C*. *reticulata* gene pools. The large number of the selected polymorphisms in heterozygosity and the heterozygosity distribution over the nine chromosomes make our GBS approach very useful for high-density genetic mapping of the considered germplasm, including for the representatives of mandarin and pummel displaying lower heterozygosity. The higher density of markers in genic areas of the genome where cross-overs are more frequent [60] is also a favorable situation for fine genetic mapping, for QTL analysis and for genetic association studies based on linkage disequilibrium.

### A validated pipeline for DP identification and a large set of diagnostic markers of *C*. *maxima/C*. *reticulata* identified along the whole genome

From the WGS data of four pummelos, three mandarins, sweet and sour oranges, “Afourer” tangor, and clementine, mapped on the reference sequence genome of clementine, Wu et al. [15] identified diagnostic polymorphisms of *C*. *reticulata* and *C*. *maxima* and inferred the “phylogenomic karyotypes” of the considered accessions. Curk et al. [17] searched diagnostic markers of the four ancestral species of cultivated *Citrus* (*C*. *reticulata*, *C*. *maxima*, *C*. *medica*and *C*. *micrantha*) from 454 amplicon sequencing data of 57 nuclear gene fragments and inferred the global phylogenomic constitution of modern varieties. Both studies demonstrated that some mandarin germplasm generally considered as truly representative of *C*. *reticulata* species was introgressed by *C*. *maxima* gene fragments. These introgressions complicate the search for polymorphisms diagnostic of *C*. *reticulata/C*. *maxima* differentiation. In our study, the introgressed areas in the genome of mandarin and pummelo representatives were identified, based on an analysis of the patterns of heterozygosity and similarity with centroids of mandarins and pummelos along the genomes. After elimination of these areas at individual level, the corrected allelic frequencies of *C*. *reticulata* and *C*. *maxima* were used to compute the differentiation parameter G_ST_ for all the markers and to select 11,133 polymorphic positions with G_ST_>0.9 as diagnostic markers. For the varieties previously analyzed from WGS data [15], the estimation of the percentage of *C*. *maxima* and *C*. *reticulata* genome contributions and the ‘phylogenomic karyotypes’ based on WGS data and our GBS analysis provided very close results. Our GBS analysis pipeline for DP identification and phylogenomic inference along the genome was therefore validated. Due to a lower density of DPs, the GBS analysis resulted in more genomic areas of undetermined phylogenetic origin, particularly for genotypes with low coverage, and therefore with a high level of missing data. Small introgressed areas can also be missed, as observed for the “Ponkan” mandarin comparison with Wu et al. [15]. The reticulate evolution of the considered germplasm and a limited number of interspecific meioses, producing interspecific mosaic genomes of large fragments, is clearly a key for the successful application of GBS to decipher phylogenomic karyotypes. This situation can also be found in the acid citrus lime and lemon groups derived from four ancestral taxa [17, 61] and GBS could be powerful in deciphering their phylogenomic structures along the genome at a lower cost than WGS. Our discovery panel to identify DSNPs of *C*. *maxima* and *C*. *reticulata* was wider than in the study by Wu et al. [15] based on WGS data. The diagnostic value of these polymorphisms is therefore greater. Their distribution along the genome is also much broader than the DSNPs identified by Curk et al. [17] from amplicon sequencing. This referential of DPs of *C*. *maxima/C*. *reticulata* will be very useful for further GBS studies, with the same protocol, on segregant progenies and extended germplasm of small citrus varieties to analyse phenotype/genotype association from a phylogenomic perspective. The 9,955 DSNPs identified in our study could also be easily converted into efficient competitive allele-specific PCR markers [17] or integrated in genotyping arrays [62] to perform large phylogenomic studies on citrus germplasm and segregating progenies, as well as studies on the impact of interspecific mosaic genomes on meiosis at diploid and tetraploid levels [63].

### Mandarins, mandarin hybrids, tangors, tangelos, orangelos and grapefruits display mosaics of large fragments of *C*. *reticulata* and *C*. *maxima* genomes

All the analyzed mandarins displayed introgressions of *C*. *maxima*. They were very limited for “Cleopatra” and “Sunki” mandarins (less than 1%), with only one introgression site identified on chromosome 3 and limited in “Ladu” mandarin (2.6% on chromosomes 2, 3 and 8) and “Szibat” mandarin (2.9% onchromosomes 2, 4 and 8). “King” and “Satsuma” mandarins displayed considerable *C*. *maxima* introgression (close to 22%) including some homozygous *C*. *maxima* regions. These results tallied with those of the study by Curk et al. [17] with a limited set of 105 diagnostic SNPs of the four citrus ancestral taxa. Indeed they found no introgression for “Cleopatra” and “Sunki”, limited introgression in “Ladu” and “Szibat”, while they observed agreater *C*. *maxima* contribution for “Satsuma” and “King” mandarins. None of the 105 markers of Curk et al. [17] were located in the small introgressed area identified by GBS for “Sunki” and “Cleopatra” and therefore they could not detect this introgression. However, this introgression was also described by Curk [64] for “Cleopatra” in a WGS study including representative of the four citrus ancestral species (“Sunki” was not part of this study). Such limited introgression implied backcrossing on *C*. *reticulata* germplasm over several generations. Statistically, after n backcross (BC) generation the remaining proportion of introgressed genome is 0.5^n+1^. Therefore after six BCs, the introgressed portion of the genome should be less than 1% (0.78%) as observed in “Cleopatra” and “Sunki”. Considering all our mandarin references, nine (including “Sunki” and “Cleopatra”) over 11 share a *C*. *maxima* introgression at chromosome 3: 3.5 to 5.5 Mb. The complete sizes of the introgressions vary between the mandarin varieties revealing different interspecific recombination locations. The shared introgressed fragments include 262 genes (https://phytozome.jgi.doe.gov/pz/portal.html#!info?alias=Org_Cclementina). It is probable that a positive selection for the *C*. *maxima* allele, of at least one of these genes, favored the conservation of linked *C*. *maxima* genome fragments over numerous backcrosses on *C*. *reticulata* germplasm.

The current results confirm, in a wider range of mandarins, that cultivated varieties previously considered as pure mandarins (*C*. *reticulata*) display introgressions of *C*. *maxima* as previously shown for “Ponkan”, “Willow leaf” and “Huanglingmiao” mandarins from WGS data [15]. Therefore, the reticulation event(s) between *C*. *reticulata* and *C*. *maxima* and further introgression processes appear to be important components of mandarin domestication. Further WGS studies will be necessary to determine whether all edible mandarins result from one or more reticulation events and how subsequent hybridization produced the modern mandarin displaying only limited parts of the *C*. *maxima* genome.

The small introgressed area of *C*. *reticulata* on chromosome 2 of “Chandler” pummelo described by Wu et al. [15] and Curk [64] from WGS data was also observed in our GBS analysis. The “Kao Pan” pummelo displayed the same introgression. “Kao Pan” is native from Thailand while Chandler was obtained in California from the cross of two Thai pummelo (“Siamese sweet” x “Siamese Pink”). Relatedness between these Thai pummelos should explain that they share the same *C*. *reticulata* introgression. These genomic structures with only one small introgression should results from successive BC on *C*. *maxima* germplasm after a reticulation event. The other pummelos appeared as pure *C*. *maxima* without identified introgression. As suggested by Wu et al. [15] and Curk et al. [17] the modern pummelo accessions appear to be good representatives of the *C*. *maxima* species, with very little or no interspecific introgression.

The origin of grapefruit (*C*. *paradisi*) is attributed to natural hybridization between pummelo and sweet orange [7, 16, 19]. This hybridization may have occurred in the Caribbean more than 200 years ago [2, 24, 65]. In our study, grapefruits had an intermediary position between the sweet orange and pummelo gene pool in the factorial analysis representation. Their “phylogenonomic karyotype” displayed a succession of large fragments of *C*. *maxima* homozygosity and *C*. *maxima/C*. *reticulata* heterozygosity in full agreement with the hypothesis of pummelo x sweet orange hybridization. Indeed, all homozygous *C*. *reticulata* areas of sweet orange are in interspecific heterozygosity in grapefruits and the small *C*. *maxima* fragment of C8 homozygous in sweet orange is also homozygous in grapefruits. The “Triumph” and “Jackson” varieties are suspected to be natural orangelos (“Jackson” being a spontaneous seedless bud-sport of “Triumph”). These varieties recently attracted great interest from the citrus industry due to a good level of tolerance to Huanglongbing (the worst citrus disease worldwide, due to the phloem bacterium*Liberobacter* sp.) compared with grapefruits [66]. The “phylogenomic karyotypes” of both varieties display large similarities with that of the grapefruits. Their “phylogenomic karyotypes” are compatibles with the orangelo hypothesis but not with a self-fertilisation of grapefruit or a pummelo by grapefruit hybridization because they display *C*. *reticulata/C*. *maxima* heterozygous genomic regions that are in *C*. *maxima* homozygosity in grapefruits.

During the 20th century, sexual hybridizations between mandarins, but also between mandarins and sweet oranges (tangors), and mandarins and grapefruits (tangelos), were carried out for “small citrus” breeding [18]. All these recent hybrids, as well as assumed natural tangors such as “Ellendale”, “Ortanique”, “Murcott”, “Temple”, “Ambersweet”, “Carvalhal”, and clementine, display a very large range of “phylogenomic karyotype” patterns with large fragments in *C*. *reticulata* or *C*. *maxima* homozygosity and *C*. *reticulata*/*C*. *maxima* heterozygosity, even though the *C*. *reticulata* contribution appears predominant for most varieties. This substantial diversity of interspecific mosaic patterns suggests frequent interspecific recombinations, favorable for genetic association studies based on the phylogenomic structures of this germplasm. Indeed, previous analysis of phenotypic diversity organization has shown that the allopatric evolution of the ancestral taxa of cultivated *Citrus* and particularly *C*. *maxima* and *C*. *reticulata* is a key component of citrus phenotypic diversity [24–27]. It is therefore highly probable that the phylogenomic patterns drive the main part of the phenotypic diversity organization of this germplasm.

## Supporting information

S1 Fig Distribution of missing data among markers and individuals. (PDF)

S2 Fig Comparison of G_ST_ distributions between the representative sets of *C*. *reticulata* and *C*. *maxima* based on initial data (G_ST1_) and data after elimination of identified introgressed areas (G_STret-max_). (PDF)

S1 Table List of plant material. (PDF)

S2 Table Chromosomal location, individual genotyping and *ret/max* G_ST_ for the 11,133 selected DPs. (XLSX)

S3 Table Percentage contribution of *C*. *maxima* and *C*. *reticulata* to the 55 analyzed varieties. (PDF)

S1 Appendix The distribution of heterozygosity and its relation with intra and interspecific variability; implication for introgression identification and genetic mapping. (PDF)
