# Supplementary material for: Genotyping by sequencing reveals the interspecific C. maxima / C. reticulata admixture along the genomes of modern citrus varieties of mandarins, tangors, tangelos, orangelos and grapefruits
Source: PLoS One. 2017 Oct 5;12(10):e0185618. doi: 10.1371/journal.pone.0185618 (PMC5628881; doi:10.1371/journal.pone.0185618)
Supplement: S1 Fig — (PDF) [file pone.0185618.s001.pdf]

Supplementary material

Amel Oueslati, Amel Salhi-Hannachi, François Luro, Hélène Vignes, Pierre Mournet and Patrick Ollitrault. Genotyping By Sequencing reveal the interspecific C. maxima / C. reticulata admixture along the genomes of modern citrus varieties of mandarins, tangors, tangelos, orangelos and grapefruits. Plos One (submitted)

S1 fig: Distribution of missing data among markers (a) and individuals (b)

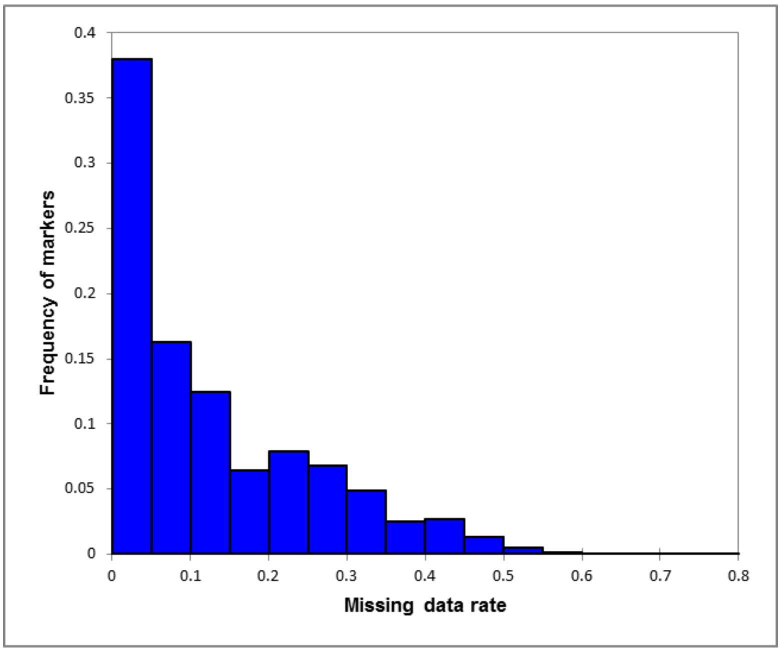

Fig S1A: distribution of missing data rate for markers

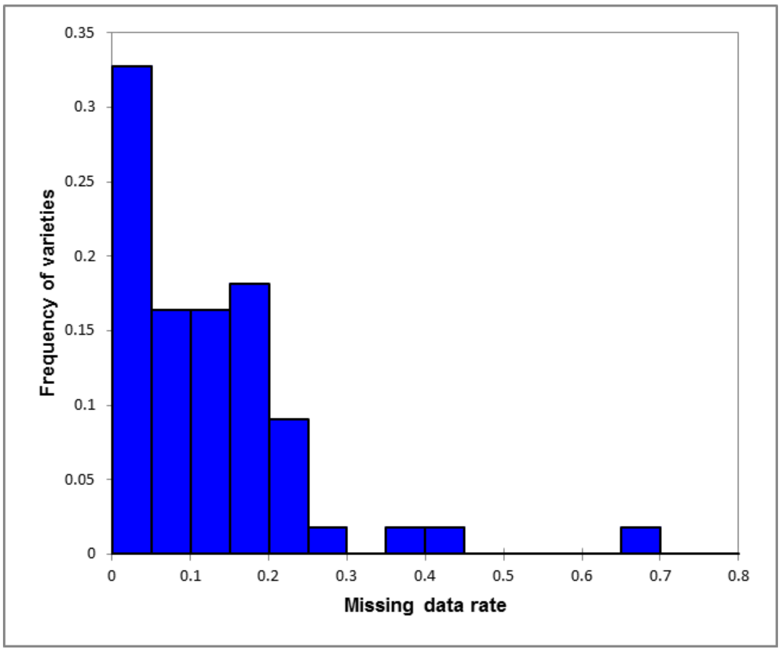

Fig S1B: distribution of missing data rate for varieties
