# Supplementary material for: Genotyping by sequencing reveals the interspecific C. maxima / C. reticulata admixture along the genomes of modern citrus varieties of mandarins, tangors, tangelos, orangelos and grapefruits
Source: PLoS One. 2017 Oct 5;12(10):e0185618. doi: 10.1371/journal.pone.0185618 (PMC5628881; doi:10.1371/journal.pone.0185618)

Supplementary material

Amel Oueslati, Amel Salhi-Hannachi, François Luro, Hélène Vignes, Pierre Mournet and Patrick Ollitrault. Genotyping By Sequencing reveal the interspecific *C. maxima / C. reticulata* admixture along the genomes of modern citrus varieties of mandarins, tangors, tangelos, orangelos and grapefruits. Plos One (submitted)

Figure S2: Comparison of GST distribution between the representative sets of *C. reticulata* and *C. maxima* based on initial data (GST1) and data after elimination of identified introgressed areas (GSTret-max)

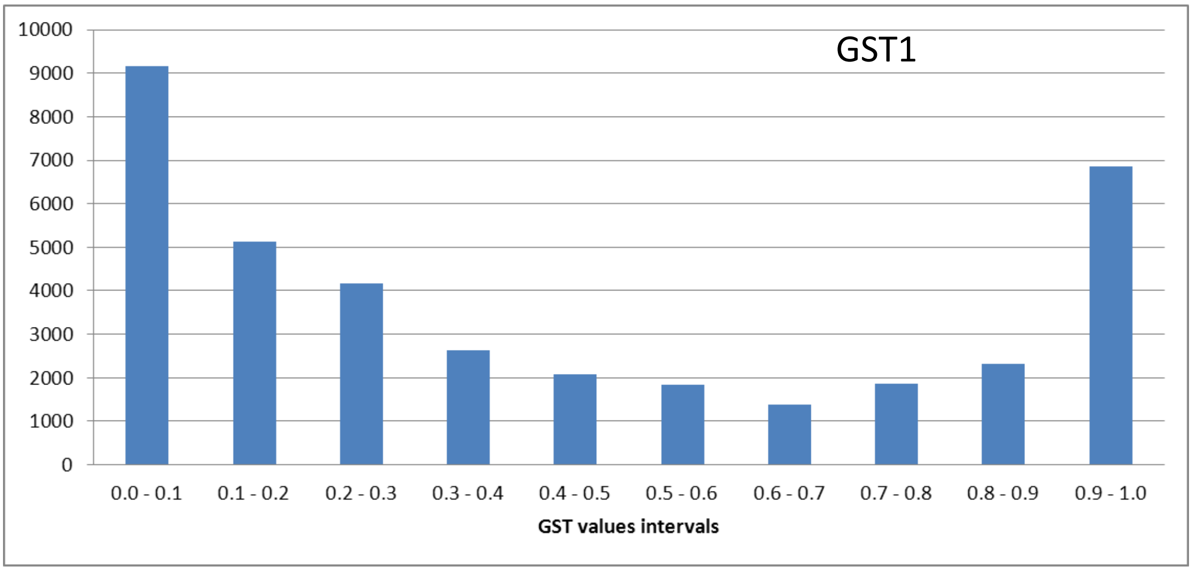

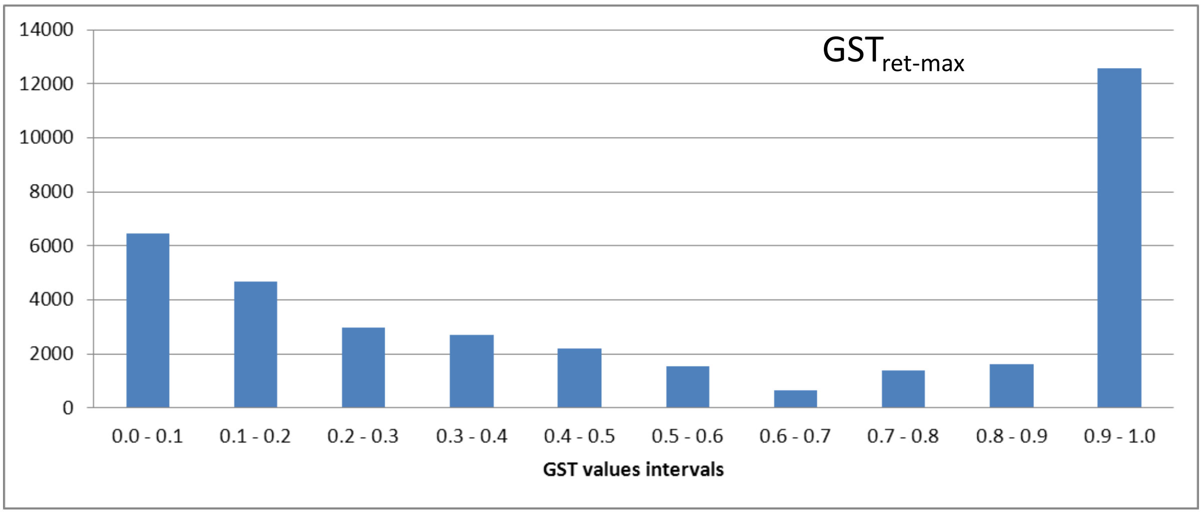

Supplement: S2 Fig — (PDF) [file pone.0185618.s002.pdf]
