# Supplementary material for: Genotyping by sequencing reveals the interspecific C. maxima / C. reticulata admixture along the genomes of modern citrus varieties of mandarins, tangors, tangelos, orangelos and grapefruits
Source: PLoS One. 2017 Oct 5;12(10):e0185618. doi: 10.1371/journal.pone.0185618 (PMC5628881; doi:10.1371/journal.pone.0185618)
Supplement: S1 Table — (PDF) [file pone.0185618.s003.pdf]

Supplementary material

Amel Oueslati, Amel Salhi-Hannachi, François Luro, Hélène Vignes, Pierre Mournet and Patrick Ollitrault. Genotyping By Sequencing reveal the interspecific C. maxima / C. reticulata admixture along the genomes of modern citrus varieties of mandarins, tangors, tangelos, orangelos and grapefruits.
Plos One (submitted)

Table S1: List of the analysed plant material

Commun name	Horticultural group	Parents in case of recent hybrids	Latin name (Swingle and Reece, 1967)	SRA Ref
Chandler pummelo	pummelo		*C. maxima*	SRA 608
Deep red pummelo	pummelo		*C. maxima*	SRA 757
KaoPan pummelo	pummelo		*C. maxima*	SRA 321
Pink pummelo	pummelo		*C. maxima*	SRA 322
Tahiti pummelo	pummelo		*C. maxima*	SRA 727
Timor pummelo	pummelo		*C. maxima*	SRA 707
Chios mandarin	mandarin		*C. reticulata*	ICVN 0100598
Cleopatra mandarin	mandarin		*C. reticulata*	ICVN 0110066
Dancy mandarin	mandarin		*C. reticulata*	SRA 594
Fuzhu mandarin	mandarin		*C. reticulata*	ICVN 0100775
King mandarin	mandarin		*C. reticulata*	SRA 583
Ladu mandarin	mandarin		*C. reticulata*	SRA 595
Ponkan mandarin	mandarin		*C. reticulata*	SRA 584
San Hu Hong Chu mandarin	mandarin		*C. reticulata*	SRA 769
Satsuma Owari mandarin	mandarin		*C. reticulata*	SRA 221
Sunki mandarin	mandarin		*C. reticulata*	ICVN 0110078
Szibat mandarin	mandarin		*C. reticulata*	SRA 596
Carvalhal mandarin hybrid	mandarin hybrid	Natural hybrid	*C. reticulata*	SRA 271
Kara mandarin	mandarin hybrid	King mandarin x Satsuma mandarin	*C. reticulata*	SRA241
Wilking mandarin	mandarin hybrid	Willow leaf mandarin x King mandarin	*C. reticulata*	SRA112
Fortune mandarin	mandarin hybrid	Clementine x Dancy mandarin	*C. reticulata*	SRA 31
Fremont mandarin	mandarin hybrid	Clementine x Ponkan mandarin	*C. reticulata*	SRA 147
Dweet tangor	tangor	Dancy mandarin x Sweet orange	*C. reticulata x C. sinensis*	ICVN0100464
Ellendale tangor	tangor	natural hybrid	*supposed C. reticulata x C. sinensis*	SRA 656
Kiyomi tangor	tangor	Miyagawa-wase (Satsuma mandarin) X Trovita orange	*C. reticulata x C. sinensis*	SRA 704
Murcott tangor	tangor	Natural hybrid	*supposed C. reticulata x C. sinensis*	ICVN 0100601
Ortanique tangor	tangor	Natural hybrid	*supposed C. reticulata x C. sinensis*	SRA 110
Temple tangor	tangor	Natural hybrid	*supposed C. reticulata x C. sinensis*	SRA 280
Ambersweet tangor	tangor x tangelo x orange	Complex hybrid	*(C. reticulata x C. sinensis) x (C. reticulata x C. paradisi) x C. si*	SRA 875
Allspice tangelo	tangelo	Imperial grapefruit x Willow leaf mandarin	*C. paradisi x C. reticulata*	SRA 327
Mapo tangelo	tangelo	Duncan grapefruit x Willow leaf mandarin	*C. paradisi x C. reticulata*	SRA 450
Minneola tangelo	tangelo	Duncan grapefruit x Dancy mandarin	*C. paradisi x C. reticulata*	SRA 451
Orlando tangelo	tangelo	Duncan grapefruit x Dancy mandarin	*C. paradisi x C. reticulata*	SRA 46
Pearl tangelo	tangelo	Willow leaf mandarin x Imperial grapefruit	*C. paradisi x C. reticulata*	SRA 296
Sampson tangelo	tangelo	Grapefruit x Dancy mandarin	*C. paradisi x C. reticulata*	ICVN0100456
San Jacinto tangelo	tangelo	Natural hybrid	*C. reticulata x C. paradisi*	SRA 297
Seminole tangelo	tangelo	Duncan grapefruit x Dancy mandarin	*C. paradisi x C. reticulata*	SRA 298
Sunrise tangelo	tangelo	Duncan grapefruit x Dancy mandarin	*C. paradisi x C. reticulata*	SRA 457
Sunshine tangelo	tangelo	Duncan grapefruit x Dancy mandarin	*C. paradisi x C. reticulata*	ICVN0100458
UGLI® tangelo	tangelo	Natural hybrid	*supposed C. reticulata x C. paradisi or C. paradisi x C. reticula*	SRA 950
Webber tangelo	tangelo	Duncan grapefruit x Dancy mandarin	*C. paradisi x C. reticulata*	ICVN0100475
Fairchild tangelo hybrid	tangor x tangelo	Clementine x Orlando tangelo	*C. reticulata x (C. paradisi X C. reticulata)*	SRA 30
Nova tangelo hybrid	tangor x tangelo	Clementine x Orlando tangelo	*C. reticulata x (C. paradisi X C. reticulata)*	SRA 158
Osceola tangelo hybrid	tangor x tangelo	Clementine x Orlando tangelo	*C. reticulata x (C. paradisi X C. reticulata)*	SRA 48
Page tangelo hybrid	tangor x tangelo	Clementine x Minneola tangelo	*C. reticulata x (C. paradisi X C. reticulata)*	SRA 159
Robinson tangelo hybrid	tangor x tangelo	Clementine x Orlando tangelo	*C. reticulata x (C. paradisi X C. reticulata)*	SRA 47
Fallglo tangelo hybrid	(tangor x tangelo) x tangor	(Clementine x Orlando) x Temple	*C. reticulata*	IVIA 466
Wekiwa tangelo hybrid	tangelo x grapefruit	Sampson tangelo x grapefruit	*C. reticulata x C. paradisi X C. paradisi*	SRA 462
Jackson orangelo	orangelo	Natural hybrid	*supposed C. sinensis x C. paradisi*	SRA 472
Triumph orangelo	orangelo	Natural hybrid	*supposed C. sinensis x C. paradisi*	ICVN0100474
Marsh grapefruit	grapefruit		*C. paradisi*	SRA 188
Star Ruby grapefruit	grapefruit		*C. paradisi*	ICVN 0100293
Bigaradier Maroc sour orange	sour orange		*C. aurantium L.*	ICVN0110033
Clemenules clementine	clementine (tangor)	Natural hybrid	*C. reticulata*	ICVN 0100389
Washington Navel Sweet orange	sweet orange		*C. sinensis*	SRA 203
