# Supplementary material for: Genotyping by sequencing reveals the interspecific C. maxima / C. reticulata admixture along the genomes of modern citrus varieties of mandarins, tangors, tangelos, orangelos and grapefruits
Source: PLoS One. 2017 Oct 5;12(10):e0185618. doi: 10.1371/journal.pone.0185618 (PMC5628881; doi:10.1371/journal.pone.0185618)
Supplement: S3 Table — (PDF) [file pone.0185618.s005.pdf]

Supplementary material

Amel Oueslati, Amel Salhi-Hannachi, François Luro, Hélène Vignes, Pierre Mournet and Patrick Ollitrault. Genotyping By Sequencing reveal the interspecific C. maxima / C. reticulata admixture along the genomes of modern citrus varieties of mandarins, tangors, tangelos, orangelos and grapefruits. Plos One (submitted)

S3 table: percentage of *C. maxima* and *C. reticulata* contribution to the 55 analyzed varieties based on 11133 DPs.

	C. reticulata	*C. maxima*
Chandler pummelo	0.2%	99.8%
Deep red pummelo	0.0%	100.0%
KaoPan pummelo	0.2%	99.8%
Pink pummelo	0.0%	100.0%
Tahiti pummelo	0.0%	100.0%
Timor pummelo	0.0%	100.0%
Chios mandarin	93.8%	6.2%
Cleopatra mandarin	99.2%	0.8%
Dancy mandarin	93.3%	6.7%
Fuzhu mandarin	93.3%	6.7%
King mandarin	78.1%	21.9%
Ladu mandarin	97.5%	2.5%
Ponkan mandarin	90.3%	9.7%
San Hu Hong Chu mandarin	96.9%	3.1%
Satsuma Owari mandarin	78.7%	21.3%
Sunki mandarin	99.1%	0.9%
Szibat mandarin	97.3%	2.7%
Kara mandarin	81.7%	18.3%
Wilking mandarin	86.2%	13.8%
Carvalhal mandarin hybrid	78.6%	21.4%
Fortune mandarin	79.0%	21.0%
Fremont mandarin	82.4%	17.6%
Clemenules clementine	81.5%	18.5%
Dweet tangor	69.8%	30.2%
Ellendale tangor	82.6%	17.4%
Kiyomi tangor	64.2%	35.8%
Murcott tangor	82.7%	17.3%
Ortanique tangor	69.6%	30.4%
Temple tangor	78.9%	21.1%
Ambersweet tangor	58.5%	41.5%
Allspice tangelo	62.0%	38.0%
Mapo tangelo	70.4%	29.6%
Minneola tangelo	73.2%	26.8%
Orlando tangelo	69.8%	30.2%
Pearl tangelo	60.6%	39.4%
Sampson tangelo	67.4%	32.6%
San Jacinto tangelo	71.4%	28.6%
Seminole tangelo	70.3%	29.7%
Sunrise tangelo	73.1%	26.9%
Sunshine tangelo	64.9%	35.1%
UGLI® tangelo	61.9%	38.1%
Webber SG tangelo	64.2%	35.8%
Fairchild tangelo	79.8%	20.2%
Nova tangelo	73.1%	26.9%
Osceola tangelo	80.5%	19.5%
Page tangelo	72.1%	27.9%
Robinson tangelo	82.2%	17.8%
Fallglo mandarin	77.7%	22.3%
Wekiwa SG tangelo	59.2%	40.8%
Jackson orangelo	36.9%	63.1%
Triumph orangelo	36.9%	63.1%
Marsh grapefruit	37.1%	62.9%
Star Ruby grapefruit	36.9%	63.1%
Bigaradier Maroc sour orange	53.1%	46.9%
Washington Navel Sweet orange	58.9%	41.1%
