# Supplementary material for: Genotyping by sequencing reveals the interspecific C. maxima / C. reticulata admixture along the genomes of modern citrus varieties of mandarins, tangors, tangelos, orangelos and grapefruits
Source: PLoS One. 2017 Oct 5;12(10):e0185618. doi: 10.1371/journal.pone.0185618 (PMC5628881; doi:10.1371/journal.pone.0185618)
Supplement: S1 Appendix — (PDF) [file pone.0185618.s006.pdf]

S1 Appendix: The distribution of heterozygosity and its relation with intra and interspecific variability; implication for introgression identification and genetic mapping.

Heterozygosity distribution and introgression identification

The search for DPs is based on the approach developed by Wu et al. [15] from WGS re-sequencing data. The goal is to identify polymorphisms differentially fixed between the two ancestral species and then to use these DPs to decipher the admixture between *C. maxima* and *C. reticulata* in modern varieties. This analysis is complicated by the fact that we are not working with real ancestors but actual varieties resulting from the domestication process and recent studies have revealed interspecific introgressions in varieties previously considered as pure *C. reticulata* or pure *C. maxima* [15, 17]. Therefore, the selection of DPs requires in a first step identifying and removing such introgressed areas for the varieties used as references, in order to have a better estimation of the differentiation parameter between the two ancestral taxa (GSTret-max). The identification of Interspecific introgressions in the varieties representative of mandarins and pummelos was based on the analysis of the pattern of two parameters along the genome: the heterozygosity (Ho) and the similarity of the considered variety with the centroid of mandarins and pummelo representative sets. Heterozygosity pattern discontinuity was a key for identifying introgressions in the genomes of basic taxa in the Wu et al. [15] study. These discontinuities of heterozygosity in admixed varieties were directly linked with the average differentiation between varieties at intra and inter-specific level. They distinguished two distinct features in the nucleotide heterozygosity distribution: one averaging ~6 het sites/kb corresponding to intraspecific heterozygosity and the other ~17 het sites/kb corresponding to interspecific *C. reticulata/ C. maxima* heterozygosity.

For GBS data, we analyzed the heterozygosity in genomic windows covering successive set of 100 polymorphic positions along the genomes. An example of distribution of these average heterozygosities is given below (Figure1) for the King mandarin. Smallest simplified histograms for all varieties are given in Figure 2.

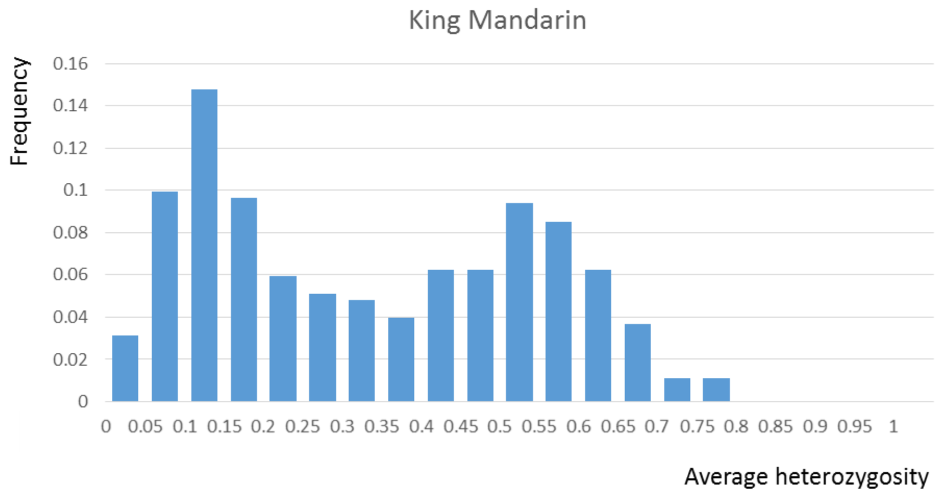

Figure 1: Distribution of the average heterozygosity (based on windows covering successive set of 100 polymorphic positions along the genome) for all analysed varieties.

Supplementary material

Amel Oueslati, Amel Salhi-Hannachi, François Luro, Hélène Vignes, Pierre Mournet and Patrick Ollitrault. Genotyping By Sequencing reveal the interspecific *C. maxima / C. reticulata* admixture along the genomes of modern citrus varieties of mandarins, tangors, tangelos, orangelos and grapefruits. Plos One (submitted)

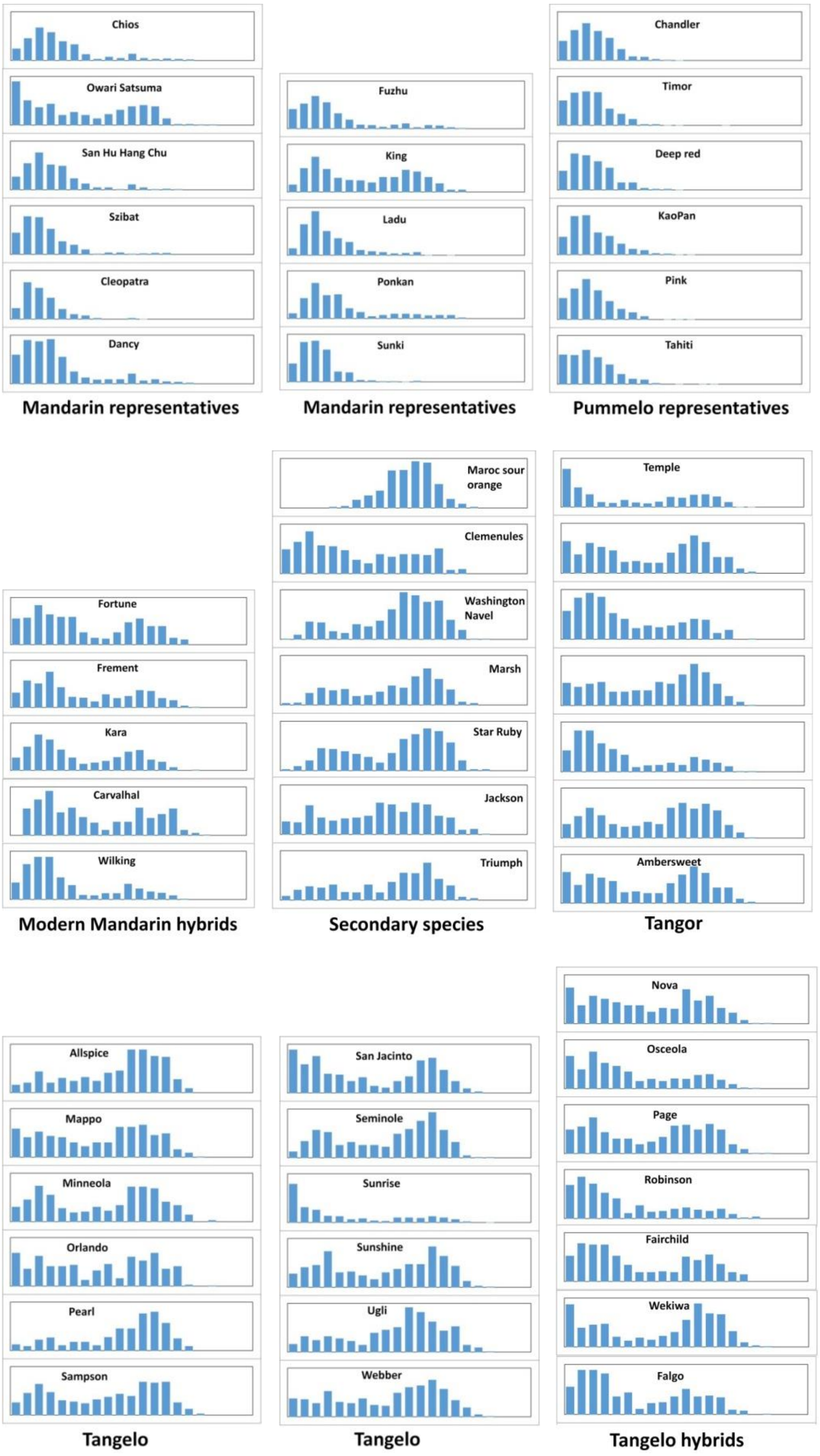

Figure 2: Distribution of the average heterozygosity (based on windows covering successive set of 100 polymorphic positions along the genome) for all analysed varieties.

Supplementary material

Amel Oueslati, Amel Salhi-Hannachi, François Luro, Hélène Vignes, Pierre Mournet and Patrick Ollitrault. Genotyping By Sequencing reveal the interspecific *C. maxima / C. reticulata* admixture along the genomes of modern citrus varieties of mandarins, tangors, tangelos, orangelos and grapefruits. Plos One (submitted)

Table: Number of heterozygous loci over the 39083 polymorphic positions and observed heterozygosity (He) for each variety; (Ci: chromosome i).

	C1	C2	C3	C4	C5	C6	C7	C8	C9	Total	He
Chandler pummelo	433	712	862	407	862	462	447	728	603	5516	0.154
Deepred pummelo	355	575	712	404	722	373	325	564	718	4748	0.152
Kao Pan pummelo	384	597	960	392	812	424	528	718	635	5450	0.156
Pink pummelo	391	455	1073	440	899	438	402	690	678	5466	0.151
Tahiti pummelo	271	543	643	298	552	314	308	509	720	4158	0.150
Timor pummelo	472	677	822	378	759	436	540	731	594	5409	0.146
Chios mandarin	463	735	1736	903	920	1270	557	1042	734	8360	0.217
Cleopatra mandarin	570	508	1042	468	687	377	389	453	616	5110	0.135
Dancy mandarin	557	1289	1223	467	767	816	392	737	524	6772	0.202
Fuzhu mandarin	593	1484	1391	573	808	933	453	821	604	7660	0.206
King mandarin	468	1229	1853	584	778	1408	989	1438	1411	10158	0.334
Ladu mandarin	553	816	1190	758	950	460	480	922	640	6769	0.182
Ponkan mandarin	590	1286	1686	565	994	1224	432	789	1391	8957	0.243
San Hu Hong Chu mandarin	703	947	916	550	821	476	522	884	1502	7321	0.193
Satsuma Owari mandarin	517	544	2961	341	1981	732	1848	1400	864	11188	0.295
Sunki mandarin	530	488	900	506	736	316	460	322	520	4778	0.140
Szibat mandarin	274	1296	887	586	658	322	459	465	606	5553	0.159
Carvalhal mandarin	486	1489	2731	1469	1911	946	414	1123	1497	12066	0.321
Clemenules Clementine	964	1498	2415	1203	1981	1292	772	932	1539	12596	0.328
Dweet tangor	911	1811	2226	1895	1655	751	1353	1292	694	12588	0.383
Ellendale tangor	921	964	1457	423	1649	301	853	706	1373	8647	0.283
Kiyomi tangor	976	1626	2802	1156	2238	630	1200	945	1680	13253	0.396
Murcot ttangor	454	683	1367	727	807	994	416	1757	1507	8712	0.266
Ortanique tangor	803	1369	1828	1474	1751	1610	1599	939	1390	12763	0.402
Temple tangor	883	1189	2362	788	908	1584	628	812	1408	10562	0.301
Allspice tangelo	655	2013	3079	1770	1506	1638	1654	915	1937	15167	0.457
Mapo tangelo	879	1393	1665	746	1051	443	973	992	968	9110	0.374
Minneola Tangelo	404	1995	2059	1157	1755	982	1717	1727	1411	13207	0.401
Orlando tangelo	582	1520	1776	1411	1868	1404	1686	954	1293	12494	0.371
Pearl tangelo	502	2393	2892	1983	2674	1841	1826	1327	1862	17300	0.481
Sampson tangelo	384	1965	2862	1525	1691	815	1243	1641	1831	13957	0.404
San Jacinto tangelo	808	2372	1670	869	1540	1033	1355	1133	1257	12037	0.350
Seminole tangelo	653	2336	3407	1550	2125	624	1839	1854	1605	15993	0.428
Sunrisetangelo	1237	1457	1485	732	925	422	244	636	293	7431	0.235
Sunshine tangelo	1611	1657	1909	1280	1564	821	1061	1449	1493	12845	0.415
UGLI® tangelo	1059	847	1989	1161	1224	1063	801	924	976	10044	0.458
Webber tangelo	1474	1767	2674	1420	1519	698	742	1249	1435	12978	0.430
Fairchild tangelo	512	2099	2104	1143	1708	1203	933	1047	1396	12145	0.321
Nova tangelo	909	997	2204	1235	2231	1337	1858	1218	1479	13468	0.351
Osceola tangelo	656	1353	2002	1571	1071	628	868	871	1614	10634	0.277
Page tangelo	486	1919	2633	1095	2113	1023	918	1432	1536	13155	0.370
Robinson tangelo	439	976	2016	826	977	325	609	741	554	7463	0.254
Fallglo mandarin	656	813	1440	1018	1777	975	626	1509	849	9663	0.302
Kara mandarin	431	1636	2331	530	1081	988	1452	1784	1302	11535	0.304
Wilking mandarin	476	1407	1600	575	795	1658	452	811	1332	9106	0.253
Jackson orangelo	662	325	1035	615	682	162	539	519	589	5128	0.416
Triumph orangelo	2054	995	3688	2164	2351	509	1886	1694	1825	17166	0.455
Wekiwa tangelo	492	1220	2935	994	2548	1899	710	1735	1784	14317	0.400
Fortune mandarin	662	1911	2351	1277	1888	1219	1449	732	857	12346	0.322
Fremont mandarin	450	1409	2886	620	1615	1249	468	1202	1786	11685	0.313
Ambersweet tangor	655	1445	1994	1670	1299	1073	1389	1361	1298	12184	0.372
Marsh grapefruit	2004	1078	3014	1695	2077	1827	1747	1671	1670	16783	0.469
Star Ruby grapefruit	2158	1166	3321	1824	2192	2054	1953	1908	1862	18438	0.483
Bigaradier Maroc sour orange	2094	2483	3708	2136	2601	1985	1884	1824	2136	20851	0.544
Washington navel sweet orange	1002	1500	2742	1963	2304	1737	1670	1588	1661	16167	0.490
